# Revision of world *Sphecomyia* Latreille (Diptera, Syrphidae)

**DOI:** 10.3897/zookeys.836.30326

**Published:** 2019-04-08

**Authors:** Kevin M. Moran, Jeffrey H. Skevington

**Affiliations:** 1 Canadian National Collection of Insects, Arachnids and Nematodes, Agriculture and Agri-Food Canada, 960 Carling Avenue, Ottawa, ON K1A 0C6, Canada Canadian National Collection of Insects, Arachnids and Nematodes, Agriculture and Agri-Food Ottawa Canada; 2 Carleton University, Department of Biology, 1125 Colonel By Drive, Ottawa, Ontario K1S 5B6, Canada Carleton University Ottawa Canada

**Keywords:** *Criorhina*, description, DNA barcode, flower fly, hoverfly, identification key, new species, species group, taxonomy

## Abstract

The 16 world species of *Sphecomyia* Latreille are revised, including seven previously undescribed species (*S.cryptica* Moran, **sp. n.**, *S.hoguei* Moran, **sp. n.**, *S.interrupta* Moran, **sp. n.**, *S.oraria* Moran, **sp. n.**, *S.pseudosphecomima* Moran, **sp. n.**, *S.sexfasciata* Moran, **sp. n.**, and *S.weismani* Moran, **sp. n.**). Descriptions, redescriptions, male genitalia photographs, distribution maps, and an illustrated key for all *Sphecomyia* are presented. DNA barcode data are provided for all 16 species with a cytochrome oxidase subunit I gene tree presented and discussed. *Sphecomyia***stat. rev.** is redefined to represent the monophyletic lineage of species within subtribe Criorhinina possessing a bare, medial vitta extending ventrally from the oral margin in both sexes, a bare gena, a bare katepimeron, a scutellum with at least anterior margin densely pruinose, an anterior ventral half of vein C before crossvein h without setae, and a narrow intersection of vein R_1_ with vein C. Three species groups of *Sphecomyia* are identified: the *S.vittata* group which possess pruinose scutellar vittae, the *S.pattonii* group which lack pruinose scutellar vittae, and *S.metallica* (Bigot), a hairy bee mimic with a completely pruinose scutum. *Criorhinatsherepanovi* Violovitsh is resurrected and transferred, along with *Criorhinaaino* Stackelberg, to the genus *Sphecomyia*: *S.tsherepanovi* (Violovitsh), **comb. n.** and *S.aino* (Stackelberg), **comb. n.***Criorhinametallica* (Bigot) is designated as the senior synonym of *C.lupina* (Williston), not junior as improperly treated, and transferred to *Sphecomyia*: *S.metallica* (Bigot), **comb. n.** The species *Sphecomyiafusca* Weisman, *S.nasica* Osburn, and *S.occidentalis* Osburn are transferred to *Criorhina* Meigen: *C.fusca* (Weisman), **comb. n.**, *C.nasica* (Osburn), **comb. n.**, and *C.occidentalis* (Osburn), **comb. n.**

## Introduction

*Sphecomyia* Latreille, 1829 (Diptera, Syrphidae), wasp fly in ancient Greek, is a Holarctic genus of large, predominantly wasp mimics placed within Eristalinae: Milesiini: Criorhinina (Thompson 1972a, 1972b). Members possess the classic, anteroventrally produced face predominant throughout the subtribe Criorhinina. Little is known of their natural history outside of scattered floral and mating records. Larval habitat is unknown with larvae never illustrated or described, though it is likely similar to other Criorhinina in that they live in rot holes, decaying wood or roots. Within the Criorhinina, generic concepts are in dire need of review. Two genera, *Criorhina* Meigen, 1822 and *Sphecomyia*, are particularly in need of attention. Traditionally, bee mimic species were placed in the genus *Criorhina* and wasp mimic species in the genus *Sphecomyia*. This hypothesis has never been tested.

Penney et al. (2012) found a strong positive relationship between mimetic fidelity and body size. This supports the relaxed-selection hypothesis, suggesting that reduced predation pressure on less profitable prey species limits the selection for mimetic perfection. *Sphecomyia*, and Criorhinina in general, are large syrphids and profitable prey targets. Thus, according to this hypothesis, they likely experience intense pressure to evolve perfect mimicry which raises the possibility that these gestalts could be convergent and these genera paraphyletic. Therefore, a dedicated review of *Sphecomyia* is a necessary step in the testing of this hypothesis.

For most of *Sphecomyia*’s history, authors considered the genus more or less related to *Temnostoma* Lepeletier and Serville, 1828, another wasp mimic genus (Williston 1886; Shannon 1922; Hull 1949). Stackelberg (1930) was the first to hypothesize that a close relationship existed between *Criorhina* and *Sphecomyia* based upon the shared characteristic of an anteroventrally produced face. Thompson (1972a, 1972b) was the second to recognize a relationship between *Criorhina* and *Sphecomyia* based primarily on shared characteristics of an anteroventrally produced face, segmented aedeagus and pilose metasternum. Shatalkin (1975) provided further support for this relationship stating the genera were “remarkably similar in the structure of the hypandrium and surstyli and especially in the capsule of the aedeagus, which has well expressed lateral wings and inner lobes that are not fused.”

Much of our knowledge about *Sphecomyia* was provided by Weisman who reviewed the genus over a series of four papers (Weisman 1964, 1965, 1966a, 1966b). Before Weisman, several authors (Williston 1886; Osburn 1908; Shannon 1925; Curran 1932) described species and provided dichotomous keys for existing species.

The first of the Weisman papers (Weisman 1964) involved the description of the species *Sphecomyiafusca* Weismann, 1964. The second work (Weisman 1965) examined male genitalia of known *Sphecomyia* and provided a dichotomous key to species based upon them. In this paper, Weisman split *Sphecomyia* into two major groupings of species: the *S.occidentalis* group (including *S.fusca*, *S.nasica* Osburn, 1908, and *S.occidentalis* Osburn, 1908), characterized by the absence of a dorsal horn on the basiphallus and a keeled, laterally sclerotized phallapodeme; and the *S.vittata* group (with *S.brevicornis* Osten Sacken, 1877, *S.columbiana* Vockeroth, 1965, *S.dyari* Shannon, 1925, *S.pattonii* Williston, 1882, *S.vespiformis* (Gorski, 1852), and *S.vittata* (Wiedemann, 1830)), characterized by a dorsal horn on the basiphallus and a unkeeled, rod-shaped phallapodeme. Weisman’s third paper (Weisman 1966a) outlined species distributions and his final paper (Weisman 1966b) provided a synoptic description of the genus and species, and their taxonomic history.

In the present study we review and expand upon Weisman’s foundation. We describe seven new species of *Sphecomyia*, provide habitus and genitalia photographs for all of the species, and provide the first key to the group since Weisman (1966b).

## Materials and methods

A list of material examined is provided. For the redescribed species, the examined material is available in a supplementary file. All specimens are labelled with a unique reference number, either with their unique collection number or in the format KMMXXXX. Label data from the studied individuals were transcribed by hand into the online CNC database and can be accessed at https://cnc.agr.gc.ca/. Specimens were borrowed from the following institutions:

**AMNH** American Museum of Natural History, New York, New York, USA

**ANSP** Academy of Natural Sciences, Philadelphia, Pennsylvania, USA

**CAS** California Academy of Sciences, San Francisco, California, USA

**CNC** Canadian National Collection of Insects, Arachnids, and Nematodes, Ottawa, Ontario, Canada

**CSCA** California State Collection of Arthropods, Sacramento, California, USA

**CSUC** Colorado State University, Fort Collins, Colorado, USA

**EMEC** Essig Museum of Entomology, University of California, Berkeley, California, USA

**INHS** Illinois Natural History Survey, Champaign, Illinois, USA

**JVSPC** Jeroen Van Steenis Personal Collection

**LACM** Los Angeles County Museum of Natural History, Los Angeles, California, USA

**MEMU** Mississippi State University, Mississippi, USA

**MZH** Finnish Museum of Natural History, Helsinki, Finland

**NBMB** New Brunswick Museum, St. John's, New Brunswick, Canada

**NSPM** Nova Scotia Museum, Halifax, Nova Scotia, Canada

**RBCM** Royal British Columbia Museum, Victoria, British Columbia, Canada

**RMNH** Naturalis Biodiversity Centre, Leiden, Netherlands

**SEMC** Snow Entomological Museum, University of Kansas, Lawrence, Kansas, USA

**UBCZ** Spencer Museum, University of British Columbia, Vancouver, British Columbia, Canada

**UCDC** R.M. Bohart Museum of Entomology, University of California, Davis, California, USA

**UCRC** Entomology Research Museum, Department of Entomology, University of California, Riverside, California, USA

**USNM** National Museum of Natural History, Washington D.C., USA

**WIRC** University of Wisconsin Insect Research Center, Department of Entomology, University of Wisconsin, Madison, Wisconsin, USA

**WSU** Maurice T. James Entomological Collection, Washington State University, Pullman, Washington, USA

### Terminology, photography, measurement, and figures

Morphological terminology follows Cumming and Wood (2017). Plant systematics follows The Plant List (2013). Morphological features of Nearctic species were examined using an Olympus SZ60 and a Zeiss SteREO DiscoveryV12 stereo microscope. Whole habitus photographs of pinned specimens were taken using the base and StackShot parts of Visionary Digital Passport II system, an Olympus OM-D EM-5 Micro 4/3 camera, and a 60 mm f2.8 macro lens (equivalent to 120 mm focal length in 35 mm photography). The specimens were illuminated by a Falcon FLDM-i200 LED dome-light. Palaearctic specimens were examined and photographed using a Leica M205-C stereoscope equipped with a Leica DFC 450 module and using 0.6× (habitus) and 1.6× (genitalia) lenses. Final images were assembled using Zerene stacker (Littlefield 2018). Photographs and descriptions are not restricted to primary types and represent our species concepts as a whole.

Male genitalia were detached after relaxation of specimen in a moisture chamber and then macerated in heated lactic acid overnight before examination and photography.

Specimen measurements were taken using the Leica measurement module in Leica Application Suite and are based upon the smallest and largest specimen of each species. Body measurements represent the distance between the frons and the posterior end of tergite 4. Wing measurements represent the distance between the tegula and the apex of the wing. Measurements of antennal segments are approximations based on the mid-line of inner surface and are presented in the ratio format scape:pedicel:flagellomere. Maps include points from all specimens examined and were produced using Simplemappr (Shorthouse 2010).

In the description of type labels the contents of each label are enclosed within double quotation marks (“ ”), while italics denote handwriting, and the individual lines of data are separated by a double forward slash (//). At the end of each record, between square brackets ([]) and separated by a comma, the number of specimens and sex, the unique identifier or number and the holding institution are given.

**Figure 1.**
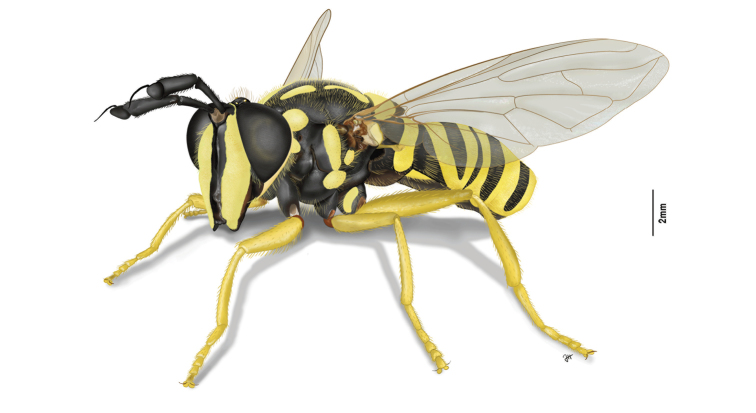
*Sphecomyiavittata* (Wiedemann, 1830).

### DNA sequencing

The right midleg was removed from selected specimens. Some legs were sent to the University of Guelph Biodiversity Institute of Ontario for sequencing of the 5¢ end of the cytochrome *c* oxidase subunit I mitochondrial gene (COI), or Barcoding region, following protocols published in (Hajibabaei et al. 2005). Others were processed in house at the Canadian National Collection of Insects (CNC) by Scott Kelso using a modified version of the same protocol with custom primers (see Table 1).

**Table 1.** Cytochrome c Oxidase I mitochondrial gene primers.

**Primer name**	**Primer design**	**Primer sequence**
Heb-F	Folmer et al. 1994	GGT CAA CAA ATC ATA AAG ATA TTG G
COI-Fx-A-R	Kelso (in prep)	CGD GGR AAD GCY ATR TCD GG
COI-Fx-B-F	Kelso (in prep)	GGD KCH CCN GAY ATR GC
COI-Fx-B-R	Kelso (in prep)	GWA ATR AAR TTW ACD GCH CC
COI-Fx-C-F	Kelso (in prep)	GGD ATW TCH TCH ATY YTA GG
COI-780R	Gibson et al. 2011	CCA AAA AAT CAR AAT ARR TGY TG

These custom primers, COI-FX-A-R, B-F, B-R, and C-F are designed to sequence the Barcoding region in three portions, labeled A, B, and C after the primers, increasing the chance of successfully sequencing heavily fragmented DNA. This enabled sampling of species for which the only available material was older than 20 years, generally considered unsuitable for barcoding.

For material sequenced at CNC, raw sequence reads were scored using Sequencer 5.4.6 (2018) and aligned together with downloaded BOLD data using MAFFT (Katoh and Standley 2013).

All sequence data obtained are stored online on the BOLD database (www.boldsystems.org). They are publicly accessible on Genbank or in the *Sphecomyia* of the World (SPHEC18) dataset available at http://www.boldsystems.org/index.php/Public_SearchTerms?query=DS-SPHEC18.

### Data analysis

Neighbor-joining, utilizing PAUP 4.0a163 (Swofford 2001) with default values, was used to explore morphological species concepts for ingroup taxa. *Blerafallax* (Linnaeus, 1758), *Milesiavirginiensis* (Drury, 1773), *Temnostomaalternans* Loew, 1864, and *Xylotaflavifrons* Walker, 1849, which also belong to Milesiini, were used as outgroups outside of Criorhinina. For outgroups inside Criorhinina, we included any described species for which we possessed a barcode.

Taxa in the tree are labeled in the following format BOLD Process ID | Taxon Name | Institution Sample ID.

## Results

### Key to *Sphecomyia* species

**Table:** 

1	Hairy-bee mimic; scutum completely pruinose (Figs 16E, 17E)	***metallica* (Bigot)**
–	Wasp mimic; scutum mostly non-pruinose with distinct regions of pruinosity (Figs 7, 10, 16A–D, 16F)	**2**
2	Thoracic scutum without pruinose vittae (Figs 7, 10); fore tarsi broadened (Fig. 6A)	**7**
–	Thoracic scutum with pruinose vittae (Figs 16A–D, F); fore tarsi not broadened (Fig. 6B)	**3**
3	Antenna not elongated, shorter than depth of head in lateral view (Fig. 5A, B)	**5**
–	Antenna elongated, longer than depth of head in lateral view (Fig. 5C)	**4**
4	Anepimeron pruinose (Fig. 19A); sternite 2 with anterior corners and lateral margins pruinose (Fig. 21D)	***vittata* (Wiedemann)**
–	Anepimeron not pruinose (Fig. 19B); sternite 2 completely black or with faint, interrupted, pruinose band anteriorly (Fig. 21E)	***vespiformis* (Gorski)**
5	Thoracic scutum with six pruinose vittae (supra-alar area pruinose in dorsal view) (Fig. 16B)	***sexfasciata* sp. n.**
–	Thoracic scutum with four pruinose vittae (supra-alar area without pruinosity) (Fig. 16A, C)	**6**
6	Scutellum completely pruinose, no black rim posteriorly (Fig. 22B); medial facial vitta interrupted by a spot of pruinosity on the tubercle (Fig. 4A); antennal segments roughly in a 3:3:2 ratio (Fig. 5A)	***interrupta* sp. n.**
–	Scutellum not completely pruinose, with black rim posteriorly (Fig. 22A); medial facial vitta entirely non-pruinose (as in Fig. 4C); antennal segments roughly in a 2:2:1 ratio (Fig. 5B)	***brevicornis* Osten Sacken**
7	Tergite 2 with a single grey, medially-interrupted band, placed medially in the tergite (Fig. 10B–D)	**14**
–	Tergite 2 with two yellow bands: the anterior interrupted, placed medially in the tergite, and the posterior, along posterior tergal margin, uninterrupted (Fig. 7)	**8**
8	Tergite 1 pruinose only in posterolateral corners (Figs 10A, 11A)	***columbiana* Vockeroth**
–	Tergite 1 with uninterrupted, pruinose band along posterior margin (Figs 7, 11B)	**9**
9	Ventral calypter with long, black pile (Fig. 20B)	***pattonii* Williston**
–	Ventral calypter with long, yellow pile (Fig. 20A)	**10**
10	Anterior two-thirds of scutellum pruinose (Fig. 22D)	***weismani* sp. n.**
–	Only anterior third or less of scutellum pruinose (Fig. 22E)	**11**
11	Scutellum completely black pilose (Fig. 15B); sternites 2–4 mostly pruinose, with shiny to dull black narrow anterior border and transverse subapical band that reaches lateral sides of sternite (Fig. 21C)	***oraria* sp. n.**
–	Scutellum at least partly yellow pilose (Fig. 15A); sternites 2–4 almost completely pruinose, with at most a triangular region of non-pruinosity posteromedially (Fig. 21A, B)	**12**
12	Sternites 2–4 with a posteromedial, triangular non-pruinose (shiny) region of the same size approximately (Fig. 21A); surstyli about twice as long as broad (Fig. 9C)	***cryptica* sp. n.**
–	Sternites 2–4 with a posteromedial, triangular non-pruinose region of different size, smaller on ensuing sternites (Fig. 21B); surstyli more than three times longer than broad (Fig. 9A, B)	**13**
13	Narrowest part of surstylus about one fourth the width of base (Fig. 9A)	***dyari* Shannon**
–	Narrowest part of surstylus about half the width of base (Fig. 9B)	***hoguei* sp. n.**
14	Cell c bare only on basal third	***pseudosphecomima* sp. n.**
–	Cell c bare on basal two-thirds	**15**
15	Ocellar triangle mostly pale pilose; silver-yellow pruinosity on face, thorax and abdomen; antennal segments pale pilose (Fig. 13A); aedeagus as in Fig. 2A	***aino* (Stackelberg)**
–	Ocellar triangle mostly dark pilose; silver-white pruinosity on face, thorax and abdomen; antennal segments black pilose (Fig. 13B); aedeagus as in Fig. 2L	***tsherepanovi* (Violovitsh)**

#### Sphecomyia stat. rev.

Taxon classificationAnimaliaDipteraSyrphidae

Figs 1 , 2A–F , 2J , 3B , 4 , 5 , 6 , 7 , 8 , 9 , 10 , 11 , 12 , 13 , 14 , 15 , 16 , 17 , 18 , 19 , 20 , 21 , 22 , 23 , 24 , 25 , 26 , 27

Sphecomye Latreille 1825: 495.
Sphecomyia Latreille in Bory 1829: 545 (also Latreille 1829: 495) – Williston 1886: 256; Osburn 1908: 14; Shannon 1925: 43; Curran 1932: 8; Stone 1965: 612; Weisman 1965: 265, 1966a: 50, 1966b: 189; Vockeroth and Thompson 1987: 736. Type species: ChrysotoxumvittatumWiedemann 1830 by subsequent designation of Macquart 1842.
Epopter Wiedemann 1830: 91. Synonymy in Evenhuis and Pont 2013: 28. Type species: Psarusornatus Wiedemann, 1830 [= Sphecomyiavittata (Wiedemann, 1830)], by monotypy.
Tyzenhausia Gorski 1852: 172. Synonymy in Wahlberg, 1854: 155. Type species: TyzenhausiavespiformisGorski 1852, by original designation.
Eurhinomallota Bigot 1882: 78. Type species: EurhinomallotametallicaBigot 1882 by original designation. Syn. n.
Eurhynomallota Bigot 1883: 225. Unjustified emendation of Eurhinomallota.
Eurinomallota Kertész 1910: 62. Unjustified emendation of Eurhinomallota.
Brachymyia Williston 1882a: 77 – Williston 1882b: 330; Shatalkin 1975: 131. Type species: Brachymyialupina Williston 1882, by original designation. Syn. n.

##### Diagnosis.

Male dichoptic. Both sexes with bare, medial vitta extending ventrally from oral margin, usually to base of antenna, except interrupted by pruinosity at facial tubercle in *S.interrupta* and only extending to facial tubercle in *S.metallica*. Gena bare. Katepimeron bare. Scutellum with at least anterior margin densely pruinose. Narrow intersection of vein R_1_ with vein C. Anterior ventral half of vein C before crossvein h without setae. Distance between apices of veins R_1_ and R_2+3_ longer than distance between apices of veins R_2+3_ and vein R_4+5_+M_1_. Abdominal pile erect. Phallapodeme banana-shaped.

##### Redescription.

**Male.** Body length: 9.2–17.1 mm. Wing length: 7.7–12.1 mm. ***Head.*** Face black, bare, concave beneath antenna, produced downwards and pruinose except with bare, medial vitta extending from oral margin, usually to base of antenna, except interrupted by pruinosity at facial tubercle in male *S.interrupta* Moran sp. n., and only extending to facial tubercle in male *S.metallica* (Bigot, 1882) or just beyond in the female; gena broad, as broad or broader than long, bare, shiny; anterior tentorial pit short, extending along ventral one-third of eye, pilose; frontal prominence distinct; frons broad, of variable size, at least partially pruinose; vertex variable in shape and pruinosity; ocellar triangle pilose, small; eye bare; male dichoptic; antenna length variable; kidney-shaped basoflagellomere, except sub-triangular in *S.brevicornis*, *S.vespiformis* and *S.vittata*, with bare arista dorsally placed.

***Thorax.*** About as long as broad, short pilose except in *Sphecomyiametallica*; postpronotum pilose; proepimeron pilose; anterior anepisternum bare, posterior anepisternum pilose; scutum with or without pruinose vittae; scutellum with at least anterior margin densely pruinose, without apical sulcus and with ventral pile fringe; katepisternum bare anteriorly, discontinuously pilose posteriorly with broadly separated patches; anepimeron with anterior portion pilose, and dorsomedial and posterior bare; katepimeron bare; metathoracic pleuron bare; without hypopleural pile at the base of the posterior thoracic spiracle; meron bare, except variable pilose in *S.vespiformis*; metathoracic spiracle about same size as flagellum; metasternum pilose; postmetacoxal bridge incomplete; plumula simple, elongate, short, not reaching calypteral margin; calypter yellow.

***Legs.*** Coxae pilose anteriorly, bare posteriorly; hind coxa pruinose anteriorly; metafemur narrow, at most slightly swollen, without basoventral setose patch; metatibia transverse apically, rounded basoventrally.

***Wing.*** Hyaline; stigmatic crossvein present; crossvein r-m at outer fourth of cell dm; anterior ventral half of vein C before crossvein h without setae (Fig. 3B); narrow intersection of vein R_1_ with vein C (Fig. 3B); distance between apices of veins R_1_ and R_2+3_ longer than distance between apices of veins R_2+3_ and R_4+5_+M_1_ (Fig. 3B); cell r_2+3_ open; vein R_4+5_ straight; vein R_4+5_+M_1_ no longer than crossvein h; vein M_2_ absent; vein CuP+CuA short, curved.

***Abdomen.*** Oval, slightly longer than broad, often with pruinose bands; abdominal pile erect.

**Figure 2.**
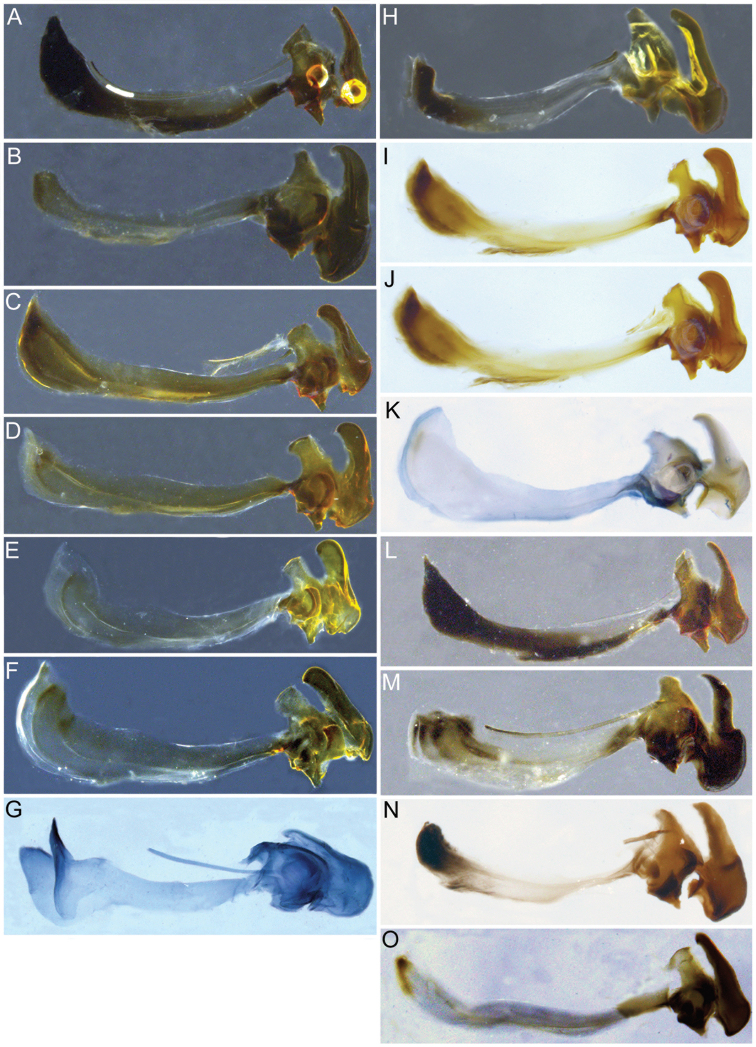
Aedaegal structure of *Sphecomyia*. **A***Sphecomyiaaino***B***Sphecomyiabrevicornis***C***Sphecomyiacolumbiana***D***Sphecomyiacryptica***E***Sphecomyiadyari***F***Sphecomyiahougei***G***Sphecomyiainterrupta***H***Sphecomyiametallica***I***Sphecomyiaoraria***J***Sphecomyiapattonii***K***Sphecomyiasexmaculata***L***Sphecomyiatsherepanovi***M***Sphecomyiavespiformis***N***Sphecomyiavittata***O***Sphecomyiaweismani*.

***Male genitalia.*** Surstyli symmetric; aedeagus segmented, with phallapodeme separated from basiphallus and distiphallus; phallapodeme banana-shaped (Fig. 2A–O); well-developed ctenidion.

##### Female.

As in male, except for usual sexual dimorphism.

##### Distribution.

13 Nearctic (12 Western, 1 Eastern) and 3 Palaearctic species.

##### Remarks.

Latreille (1825) first referenced the genus in French vernacular as *Sphecomye* based on specimens collected in Carolina by D. Bose. No description was included, nor was a specific epithet assigned to the specimens, thus the name is considered unavailable. Stark (1828) provided a translation from French vernacular as *Sphecomyia*, but as it referenced Latreille (1825) it still is not considered available. *Sphecomyia* is first made available in Latreille (1829) in which description of the genus is provided. Macquart (1842) designated *Chrysotoxumvittatum* Wiedemann as the type species by monotypy.

In this paper, *Sphecomyia* is redefined as the monophyletic unit of species within Criorhinina that possess the following characters: a bare, medial vitta extending ventrally from the oral margin in both sexes, a bare gena, a bare katepimeron, a scutellum with at least anterior margin densely pruinose, an anterior ventral half of vein C before crossvein h without setae and a narrow intersection of vein R_1_ with vein C. While the combination of characters used to define *Sphecomyia* is unique, the subtribe Criorhinina is rife with homoplasy and the presence of one or more of these character states without all the others should not be taken as an indication a species belongs in *Sphecomyia*.

*Brachymyia* Williston, 1882 and *Eurhinomallota* Bigot, 1882 are newly synonymized with *Sphecomyia* as the type species of both genera fall within this definition and are combined with it as a result of this change. This decision is further supported by molecular evidence showing a close relationship with *Sphecomyia*, i.e., the present COI gene tree and a multi-gene molecular phylogeny of the Criorhinina which will be presented in an upcoming paper. It is the authors opinion that combination with *Sphecomyia*, as opposed to resurrecting the concept as a monotypic genus, serves to emphasize its relationship with the group.

There are three major, monophyletic lineages of *Sphecomyia*. The *vittata* group, composed of the species with pruinose vittae on the scutum, i.e., S. *brevicornis*, *S.interrupta* sp. n., *S.sexfasciata* Moran sp. n., *S.vespiforme*, and *S.vittata*. Secondly, the *pattonii* group comprised of species with broadened fore tarsi and without pruinose vittae on the scutum, i.e., *S.aino* (Stackelberg, 1955), *S.cryptica* Moran sp. n., S.dyari, *S.hoguei* Moran sp. n., *S.oraria* Moran sp. n., *S.pattonii*, *S.pseudosphecomima* Moran sp. n., *S.tsherepanovi* (Violovitsh, 1973), and *S.weismani* Moran sp. n. The third group comprises only one species, *Sphecomyiametallica*, which has a completely pruinose scutum. *S.metallica* shares several characters with the *vittata* group. It has elongated surstyli, with a rounded baso-ventral lobe, reminiscent of the *vittata* group and it lacks the broadened fore tarsi of the *pattonii* group. Morphological characters of *Sphecomyia* are discussed in greater detail in the morphology section (see below).

Also of note, Shatalkin (1975) redefined *Brachymyia* as representing the species of *Criorhina* which lack a ventral scutellar fringe and possess hypopleural pile. The type of *Brachymyia*, *Sphecomyiametallica*, does not fit this generic definition as it has a ventral scutellar fringe and lacks hypopleural pile. Neither *Criorhinaberberina* (Fabricius, 1805) nor the other species Shatalkin combined with *Brachymyia* are closely related to the type. Definitions of other criorhinine genera might change after this work.

#### Sphecomyia aino

Taxon classificationAnimaliaDipteraSyrphidae

(Stackelberg, 1955) comb. n.

Figs 2A , 10B , 12B , 13A , 14B , 24

Penthesilea aino Stackelberg 1955: 347. Type locality: Russia: Far East, Sakhalin Central Experimental Station. [ZISP]
Criorrhina stackelbergi Violovitsh 1973: 112. Type locality: Russia: Siberia, Altai Mts. [ZISP]
Criorhina stackelbergi Violovitsh 1976:341 – 1982: 211, 1983: 137; Peck 1988:207.
Criorrhina aino Mutin and Barkalov 1990: 118.
Criorhina aino Mutin and Barkalov 1997: 217 – Gritskevich 1998: 11; Barsukova 2012: 187 Mutin et al. 2016: 9; Mutin and Barkalov 2016: 21.

##### Diagnosis.

Species similar to *S.pseudosphecomima* or *S.tsherepanovi* but can be distinguished by the following characters: cell c bare on basal two-thirds; ocellar triangle pale pilose; silver-yellow pruinose; basiphallus as in Fig. 2A.

##### Redescription.

**Male.** Body length: 10.0 to 13.5 mm. Wing length: 8.2 to 8.4 mm. ***Head.*** Face silver-yellow pruinose with shiny, black, medial vitta extending from oral margin to base of antenna; frons broad, about as long as broad at antenna, two-thirds as broad at vertex as at antenna, bare, with silver-yellow pruinosity along posterior rim; vertex triangular, longer than broad, shiny, with ocellar triangle pale pilose; postocular border silver-yellow pruinose; postocular pile black; occipital pile pale; male narrowly dichoptic; antenna black, pale pilose, with length of segments roughly in a 3:3:2 ratio.

***Thorax.*** Sub-shiny black; postpronotum pale pilose, scutum pale pilose, except with black pile posteromedially; scutellum, postalar callus, proepimeron pale pilose, posterior anepisternum pale pilose; posterior katepisternum pale pilose with broadly separated patches; anterior anepimeron, metasternum pale pilose; postpronotum, anterior eighth of scutellum, broad posterior margin of anepisternum and dorso-posterior corner of katepisternum silver-yellow pruinose; area between postpronota weakly silver-yellow pruinose, except shiny medially; anepimeron shiny; scutum without pruinose vittae; ventral calypter with long pale pile.

***Legs.*** Foreleg black, except reddish-yellow at apex of femur; fore tarsi slightly broadened; midleg yellow, except basal four-fifths of femur and last two tarsomeres black; hind leg reddish-yellow, except last two tarsomeres black; legs pale pilose, except black pilose on fore tibia, fore tarsi, extreme apex of fore femur and last two mid and hind tarsomeres; hind coxa silver-yellow pruinose.

***Wing.*** Hyaline; microtrichia absent from following areas: cell bc; basal two-thirds of cell c; basal fourth of cell sc; cell r_1_ from base almost to crossvein r-m; broad basal portion of cell br (before origin of M) and about basal two-fifths of narrower portion of this cell (caudad of spurious vein only); cell bm, except apex and narrow anterior and posterior margins of about apical fourth; broad anterior margin of cell cua; narrow, elongate, oval area proximal to vein A_1_.

***Abdomen.*** Tergites and sternites shiny to sub-shiny, black with silver-yellow pruinosity as follows: tergite 1 pruinose posteriorly; tergite 2 with thin, interrupted, medial band which curves posteriorly to reach the posterolateral corners; tergite 3 with thin, medial, interrupted band which does not curve anteriorly; tergite 4 with similar but thinner band; sternite 1 weakly pruinose; sternites 2 and 3 pruinose on anterior third and sub-shiny black on remainder; sternite 4 with anteromedial pruinose spots; pile of abdomen pale.

***Male genitalia.*** Surstylus not elongated, about as long as broad, curving upward ventrally; pilose on anterolateral outer surface of surstylus; minute spines on ventral surface and apical half of interior lateral surface; basal fourth of ventral surface of the surstylus produced into a lobe directed anteriorly, with minute pubescence on ventral and lateral inner surface; cerci rounded, with no invagination on posterior border; aedeagus as in Fig. 2A.

##### Female.

Similar to male except normal sexual dimorphism.

##### Distribution.

Far Eastern Russia including Sakhalin Island and westerly into Eastern Siberia (Fig. 24).

##### Biology.

Collected visiting flowers of *Cornusalba* (L.) Opiz, *Weigelamiddendorffiana* C. Koch, *Rhododendronaureum* Georgi, and *Rhododendrondauricum* L. Known to hilltop. Recorded flying in June and July.

##### Remarks.

Two morphospecies are recognized in the *Sphecomyiaaino* complex, *S.aino* from continental East Palaearctic and S. ts*herepanovi* from the Japanese and Kuril Islands. *Sphecomyiaaino* are silver-yellow pruinose with mostly pale pile on their antennal segments and ocellar triangle. *Sphecomyiatsherepanovi* are silver-white pruinose with mostly black pile on their antennal segments and ocellar triangle. Additionally, the two populations were found to possess differently shaped dorsal horn on their basiphallus (Fig. 2A, L). We argue that these character differences, along with the 3% difference in the DNA barcode between the two taxa, especially considering that the mainland population has little to no variation in COI even across distances greater than 3000 km, are significant enough to warrant separation into two distinct species.

Of note are a male and female pair of specimens collected together in mainland Russia. Both possess characters associated with *S.tsherepanovi*. They are silver-white pruinose. The female is fully black pilose on its antennal segments and ocellar triangle, while the male is mixed pale and black pilose on these regions. The basiphallus of the male is identical to that of Fig. 2A. Both were barcoded, with Folmer regions identical to *S.aino* recovered. We consider this pair aberrant and not representative of *S.aino*. The male appears teneral as its exoskeleton is light brown in color. The female has a completely black pilose scutum and face along with a mixed pale and black pilose scutellum. These characters are not seen in another specimen of either species and may be indicative of a mutation. It is also is possible the female is teneral. Lending support to this hypothesis is communication with Russian Syrphidae researcher Valery Mutin who indicates all local specimens he collected fit the typical *S.aino* morphospecies concept. Still it is possible, though unlikely, that these specimens indicate that the mainland species is more variable than we have come to believe.

#### Sphecomyia brevicornis

Taxon classificationAnimaliaDipteraSyrphidae

Osten Sacken, 1877

Figs 2B , 5B , 16A , 17A , 18A , 21G, H , 22A , 23

Sphecomyia brevicornis Osten Sacken 1877: 341 – Röder 1879: 97; Williston 1882b: 328, 1886: 258; Aldrich 1905: 404; Osburn 1907: 4, 1908: 11; Kertész 1910: 348; Cole and Lovett 1921: 293; Shannon 1925: 43; Hull 1949: 264; Stone et al. 1965: 612; Weisman 1965: 266, 1966a: 51, 1966b :193; Boyes and van Brink 1967: 432, 1970: 212; Cole and Schlinger 1969: 331; Telford 1975: 21. Type locality: Webber Lake, Sierra County, California. [MCZ]
Sphecomyia vespiformis of Curran 1932: 8, not Gorski 1852. Misidentification.

##### Diagnosis.

Species most similar to *S.interrupta* sp. n. and *S.sexfasciata* sp. n. but can be distinguished by the following characters: scutum with two pairs of pruinose vittae; cell c completely microtrichose; antenna possessing a 2:2:1 ratio of segments; frons bare; anepimeron not pruinose; anterior three-fourths of scutellum pruinose; medial facial vitta not interrupted by a spot of pruinosity.

##### Redescription.

**Male.** Body length: 11.0–16.0 mm. Wing length: 9.7–10.9 mm. ***Head.*** Face yellow pruinose with shiny, black, medial vitta extending from oral margin to base of antenna; frons broad, about as long as broad at antenna, two-thirds as broad at vertex as at antenna, bare, with yellow pruinosity along posterior third; vertex triangular, longer than broad, shiny, with ocellar triangle black pilose; postocular border yellow pruinose; postocular pile black; occipital pile yellow; male narrowly dichoptic; antenna black, black pilose, with length of segments roughly in a 2:2:1 ratio.

***Thorax.*** Matte black; postpronotum yellow pilose; scutum yellow pilose, except with black pile posteromedially; scutellum yellow pilose anteriorly and black pilose posteriorly; postalar callus, proepimeron, posterior anepisternum yellow pilose; posterior katepisternum yellow pilose with broadly separated patches; anterior anepimeron yellow pilose; metasternum yellow pilose; postpronotum, anterior three-fourths of scutellum, broad posterior margin of anepisternum and dorso-posterior corner of katepisternum yellow pruinose; anepimeron usually shiny, rarely with weak pruinosity; scutum with two pairs of pruinose vittae: anterior pair long, running from anterior edge of scutum to transverse suture; posterior pair shorter and terminating before posterior edge; ventral calypter with long yellow pile.

***Legs.*** Foreleg reddish-yellow, except basal four-fifths of femur and last three tarsomeres black; midleg reddish-yellow, except basal four-fifths of femur and last three tarsomeres black; hind leg reddish-yellow, except last two tarsomeres black; legs yellow pilose, except black pilose on last three tarsomeres; hind coxa yellow pruinose.

***Wing.*** Hyaline; microtrichia absent from following areas: broad anterior margin of cell cua.

***Abdomen.*** Tergites and sternites shiny to sub-shiny, black with yellow pruinose markings as follows: tergite 1 pruinose along posterior margin; tergite 2 with broad, interrupted, truncate medial band which meets a broad, uninterrupted posterior band in the posterolateral corners of tergite; tergite 3 with broad medial band, sometimes very narrowly interrupted, that joins with broad posterior band in two places, creating a medial diamond-shaped spot of no pruinosity; pattern on tergite 4 same as tergite 3; sternite 1 shiny; sternites 2 to 4 variable pruinose: ranging from almost completely pruinose, with a small region of non-pruinosity posteromedially to mostly pruinose, except with narrow anterior border and transverse subapical band shiny to dull black; sternites 6 to 8 pruinose; pile of abdomen yellow.

***Male genitalia.*** Surstylus elongated, about two and a half times as long as broad, apex acute, directed ventrally; pile on dorsal surface of surstylus, increasing in length posteriorly; minute spines on ventral surface and apical three-fourths of lateral inner and outer surface; basal fourth of the ventral surface of the surstylus produced into a lobe directed ventrally, with minute pubescence on ventral and lateral inner surface; cerci rounded, with invagination on posterior border; aedeagus as in Fig. 2B.

##### Female.

Similar to male except normal sexual dimorphism.

##### Distribution.

U.S.A.: Washington, Oregon, California, Idaho, and Montana. Canada: Alberta and British Columbia (Fig. 23). Extends south from southern British Columbia, as well as the southeastern corner of Alberta, through the coastal and mountainous areas of Washington state, through Oregon and into the Sierra Nevada and midcoastal regions of California. Also known from forested regions of northern Idaho and western Montana.

##### Biology.

Collected visiting flowers of *Vaccinium* L. sp., *Phacelia* Juss. sp., *Ceanothus* L. sp. and *Berberisaquifolium* Pursh. Recorded flying late April through late July, with one outlier in late August.

##### Remarks.

*Sphecomyiabrevicornis* shows intraspecific variation on sternites 2 to 4. Northern specimens (i.e. Washington, British Columbia, Idaho, Montana) possess larger non-pruinose, shiny areas on these sternites (Fig. 21G). On Californian specimens these sternites are more pruinose (Fig. 21H). In Oregon there are apparent intermediates of the two states. Californian specimens can be, but are not always, weakly pruinose on the anepimeron, as opposed to the shiny anepimeron found in most. No other morphological characters to distinguish between the two populations were found. Two barcodes for *S.brevicornis* were recovered. One from an Alberta specimen and one from a California specimen. The two were 1.3% different, however, neither barcode was complete with the Albertan one missing data at both ends of the sequence and the Californian one missing the middle B fragment. Additional and complete sequences of both the northern and southern morphotypes of *S.brevicornis* are needed to determine whether a gradient exists or whether two discrete clusters are resolved.

#### Sphecomyia columbiana

Taxon classificationAnimaliaDipteraSyrphidae

Vockeroth, 1965

Figs 2C , 10A , 11A , 12A , 14A , 22C , 26

Sphecomyia columbiana Vockeroth 1965: 86 – Weisman 1965: 268, 1966: 194; Telford 1975: 21. Type locality: 32 miles southwest Terrace, British Columbia, Canada. [CNC]

##### Diagnosis.

It can be confused with *S.cryptica* sp. n., *S.dyari*, *S.hoguei* sp. n., *S.oraria* sp. n., and *S.pattonii* but is distinguished by a tergite 1 densely pruinose only in the posterior corners.

##### Redescription.

**Male.** Body length: 13.2–14.3 mm. Wing length: 9.9–10.5 mm. ***Head.*** Face yellow pruinose with shiny, black, medial vitta extending from oral margin to base of antenna; frons broad, about as long as broad at antenna, two-thirds as broad at vertex as at antenna, bare, with silver pruinosity along posterior half; vertex triangular, longer than broad, shiny, with ocellar triangle black pilose; postocular border yellow pruinose; postocular pile black; occipital pile yellow; male narrowly dichoptic; antenna black, black pilose, with length of segments roughly in a 3:3:2 ratio.

***Thorax.*** Shiny black; postpronotum yellow pilose with occasional black pile; scutum and scutellum mostly black pilose with occasional yellow pile; postalar callus mixed black and yellow pilose; proepimeron yellow pilose; anepisternum yellow pilose posteriorly; posterior katepisternum yellow pilose with broadly separated patches; anterior anepimeron yellow pilose; metasternum yellow pilose; postpronotum, anterior eighth of scutellum, broad posterior margin of anepisternum and dorso-posterior corner of katepisternum yellow pruinose; area between postpronota weakly silver pruinose, except shiny medially; anepimeron shiny; scutum without pruinose vittae; ventral calypter with long yellow pile.

***Legs.*** Foreleg black, except extreme apex of femur and anterior fourth of tibia reddish-yellow; fore tarsi slightly broadened; midleg reddish-yellow, except basal four-fifths of femur and last two tarsomeres black; hind leg reddish-yellow, except basal four-fifths of femur and last two tarsomeres black; legs yellow pilose, except black pilose on fore tibia, fore tarsi, extreme apex of fore femur, and last two mid and hind tarsomeres; hind coxa silver pruinose.

***Wing.*** Hyaline; microtrichia absent from following areas: cell bc; basal sixth of cell c; basal fourth of cell sc; cell r_1_ from base almost to crossvein r-m; broad basal portion of cell br (before origin of M) and about basal two-fifths of narrower portion of this cell (caudad of spurious vein only); cell bm, except apex and narrow anterior and posterior margins of about apical fourth; broad anterior margin of cell cua; narrow, elongate, oval area proximal to vein A_1_.

***Abdomen.*** Tergites and sternites shiny to sub-shiny, black with yellow pruinose markings as follows: tergite 1 pruinose in posterolateral corners; tergite 2 with broad, interrupted, truncate medial band which meets a narrow, uninterrupted posterior band in the posterolateral corners of tergite; tergite 3 with similar medial band, but more narrowly interrupted; pattern on tergite 4 same as tergite 3, except medial band very narrowly or incompletely interrupted; sternite 1 shiny; sternites 2 to 4 mostly pruinose, except with narrow anterior border and transverse subapical band shiny to dull black; sternites 6 to 8 pruinose; pile of abdomen yellow, except with some black pile present on posterior halves of tergites 3 and 4 and on postabdomen.

***Male genitalia.*** Surstylus not elongated, about as long as broad, curving downward ventrally; pile on anterolateral outer surface of surstylus; minute spines on ventral surface and apical half of interior lateral surface; basal fourth of the ventral surface of the surstylus produced into a lobe directed posteroventrally, with minute pubescence on ventral and lateral inner surface; cerci rounded, with no invagination on posterior border; aedeagus as in Fig. 2C.

##### Female.

Similar to male except normal sexual dimorphism.

##### Distribution.

Canada: British Columbia. U.S.A.: Washington (Fig. 26). Known from two close localities on the central coast of British Columbia and several clustered localities in southeastern Washington.

##### Biology.

Collected visiting flowers of *Heracleummaximum* W. Bartram. Recorded flying April through June.

#### Sphecomyia cryptica

Taxon classificationAnimaliaDipteraSyrphidae

Moran sp. n.

http://zoobank.org/E96D3388-CEDE-4BB9-84A7-6F6ACEF800C7

Figs 2D , 7C , 8C , 9C , 15A , 21A , 22E , 25

Sphecomyia pattonii of authors Cole and Lovett 1921:293, not Williston 1882. Misidentification.

##### Type locality.

U.S.A.: Oregon: Klamath County, Lake of the Woods, 42.36056, −122.205, 1500 m.

##### Types.

***Holotype*** male, pinned. Original label: “Crater Lake // Nat. Park” “A. L. Lovett // Coll *8 22*” “CNC47005”. [1♂, CNC47005, CNC]

***Paratypes*** : U.S.A., California: Del Norte Co., Darlingtonia, HWY 199, Six Rivers National Forest, 41.838611, −123.946111, 120 m, F.C. Thompson, 1.vi.2009, USNM_ENT01261991; USNM_ENT01261992; USNM_ENT01261993 (3♂, USNM); M. Hauser, 1.vi.2009, KMM0891; KMM0892 (2♂, CSCA). Oregon: 12–15 miles east of Ashland, Dead Indian Road, 42.2695, −122.4388, 1371 to 1493 m, H.A. Scullen, 17.vii.1930, KMM0805 (1♀, WIRC); Anna Creek, 42.9253, −122.1727, A.L. Lovett, 22.viii, CNC47006 (1♂, CNC); Crater Lake National Park, 42.8684, −122.1685, 2133 m, E.C. Van Dyke, 17.vii.1922, KMM0913; KMM0915 (2♀, CAS); A.L. Lovett, 22.viii, CNC47004; (1♂, CNC); KMM0903; KMM0904 (2♂, AMNH); D.C. Lowrie, 21.vii.1951, USNM01261990 (1♂, USNM); KMM0902 (1♀, WSU); E.C. Van Dyke, 14.vii.1934, KMM0914 (1♂, CAS); Douglas Co., Diamond Lake, 43.1699, −122.1681, E.C. Van Dyke, 16.vii.1934, KMM0916 (1♂, CAS); Jackson Co., 1.5 miles north Mount Ashland Ski Bowl, 42.1058, −122.6994, P. Rude, 4.vii.1970, EMEC371299 (1♂, EMEC); Jackson Co., Mount Ashland, 42.08, −122.7175, 2073 m, P. Rude, 26.vii.1966, EMEC371298; EMEC371348 (2♀, EMEC); Klamath Co., Crescent Lake, 43.5084, −121.9685, E.R. Jaycox, 4.vii.1952, CNC143004 (1♀, CNC); Klamath Co., Klamath Falls, Coary Ranch, 42.225, −121.78111, *Prunusdemissa*, J. Schuh, 12.vi.1964, USNM1028902 (1♂, USNM); Klamath Co., Lake of the Woods, 42.3606, −122.205, E.C. Van Dyke, 10.vii.1934, KMM0906; KMM0907; KMM0908; KMM0909; KMM0910; KMM0911; KMM0912; KMM0917 (7♂,1♀, CAS); 42.360561, −122.205000, 1508 m, H.A. Scullen, 18.vii.1930, KMM0905 (1♂, WIRC); Klamath Co., Pelican Butte Road, 42.4633, −122.1103, P.H. Arnaud Jr., 29.vii.1967, USNM1071333 (1♂, USNM); Linn Co., Hoodoo Ski Bowl, 44.4072, −121.8719, 1402 m, P.A. Opler, 25.vii.1966, EMEC371347; EMEC371349; EMEC371350 (1♂,2♀, EMEC); Linn Co., Marion Forks, 44.6155, −121.9468, R.L. Fischner, 30.vi.1962, USNM01261994 (1♀, USNM); Mount Hood, Cloud Cap, 45.402875, −121.654162, 1520 m, M.C. Lane, 17.vii.1933, KMM0793; KMM0794 (2♂, WIRC); Mount Hood, 45.5389 -121.5681, 1524 m, M.C. Lane, 21.vi.1925, KMM0798 (1♂, WIRC); G.P. Englekardt, viii, USNM1028877; USNM1028911 (1♂,1♀, USNM); Mount Jefferson, 44.67429, −121.7990, subalpine regions, J.C. Bridwell, 20.vii.1907, CNC47015 (1♀, CNC); Timberline near Government Camp, Mount Hood, 45.3309, −121.7107, E.C. Van Dyke, 28.vii.1937, KMM0841; KMM0842 (2♀, CAS); Vidae Falls, Crater Lake National Park, 42.8844, −122.09970, B.V. Peterson, 10.vii.1968, CNC47016 (1♀, CNC).

##### Diagnosis.

Species similar to *S.columbiana*, *S.dyari*, *S.hoguei* sp. n., *S.oraria* sp. n., and *S.pattonii* but can be distinguished by the following characters: tergite 1 with uninterrupted, pruinose band along posterior margin; scutellum mixed black and yellow pilose; ventral calypter with long yellow pile; sternites 2 to 4 almost completely pruinose, with a triangular region of non-pruinosity posteromedially.

##### Description.

**Male.** Body length: 11.9–14.2 mm. Wing length: 8.9–10.7 mm. ***Head.*** Face yellow pruinose with shiny, black, medial vitta extending from oral margin to base of antenna; frons broad, about as long as broad at antenna, two-thirds as broad at vertex as at antenna, bare, with yellow pruinosity along posterior third; vertex triangular, longer than broad, shiny, with ocellar triangle black pilose; postocular border yellow pruinose; postocular pile black; occipital pile yellow; male narrowly dichoptic; antenna black, black pilose, length of segments roughly in a 3:3:2 ratio.

***Thorax.*** Sub-shiny black; postpronotum yellow pilose; scutum yellow pilose, except with black pile posteromedially; scutellum mostly yellow pilose with occasional black pile; postalar callus, proepimeron, posterior anepisternum yellow pilose; posterior katepisternum yellow pilose with broadly separated patches; anterior anepimeron yellow pilose; metasternum yellow pilose; postpronotum, anterior fourth of scutellum, broad posterior margin of anepisternum and dorso-posterior corner of katepisternum yellow pruinose; area between postpronota weakly yellow pruinose, except shiny medially; anepimeron shiny; scutum without pruinose vittae; ventral calypter with long yellow pile.

***Legs.*** Foreleg black, except extreme apex of femur and anterior third of tibia reddish-yellow; fore tarsi slightly broadened; midleg reddish-yellow, except last two tarsomeres black; hind leg reddish-yellow, except last two tarsomeres black; legs yellow pilose, except, fore tibia, fore tarsi, extreme apex of fore femur and last two mid and hind tarsomeres black pilose; hind coxa yellow pruinose.

***Wing.*** Hyaline; microtrichia absent from following areas: cell bc; cell r_1_ from base to about halfway to crossvein r-m; broad basal portion of cell br (before origin of M) and about basal two-fifths of narrower portion of this cell (caudad of spurious vein only); cell bm, except apex and narrow anterior and posterior margins of about apical fourth; broad anterior margin of cell cua; narrow, elongate, oval area proximal to vein A_1_.

***Abdomen.*** Tergites and sternites shiny to sub-shiny, black with yellow pruinose markings as follows: tergite 1 pruinose along posterior margin; tergite 2 with broad, interrupted, truncate medial band which meets a narrow, uninterrupted posterior band in the posterolateral corners of tergite; tergite 3 with similar medial band, but more narrowly interrupted; pattern on tergite 4 same as tergite 3 except medial band very narrowly or incompletely interrupted; sternite 1 shiny; sternites 2 to 4 almost completely pruinose, with a triangular region of non-pruinosity posteromedially; sternites 6 to 8 pruinose; pile of abdomen yellow, except sometimes with scattered black pile present on postabdomen.

***Male genitalia.*** Surstylus elongated, about twice as long as broad, curving upward dorsally; pile on dorsal surface of surstylus, increasing in length posteriorly; minute spines on ventral surface, with apical three-fourths of lateral inter surface also with spines; basal fourth of the ventral surface of the surstylus produced into a lobe directed anteriorly, with no minute pubescence present; cerci rounded, with invagination on posterior border; aedeagus as in Fig. 2D.

##### Female.

Similar to male except normal sexual dimorphism.

##### Distribution.

U.S.A.: Oregon (Fig. 25). Restricted to the Oregon portion of the Cascade Range.

**Etymology.** The specific epithet is derived from the Greek *kryptos* (Brown 1956: 241), which means hidden or secret and references the difficulty of distinguishing this species from *S.dyari* and *S.pattonii*.

##### Biology.

Cole and Lovett (1921) misidentified specimens Lovett collected as *S.pattonii*. Lovett noted he observed them “entirely in the forenoon, occurring just at the edge of clearings and flying swiftly, close to the ground, resting occasionally in low growing shrubbery at the very edge of dense forests”. They have been collected visiting flowers of Prunusvirginianavar.demissa (Nutt. ex Torr. and A. Gray) Torr. A specimen collected by Bridwell notes it was collected in the sup-alpine region. Recorded flying June through August.

#### Sphecomyia dyari

Taxon classificationAnimaliaDipteraSyrphidae

Shannon, 1925

Figs 2E , 7A , 8A , 9A , 21B , 25

Sphecomyia dyari Shannon 1925: 43 – Vockeroth 1965: 86; Stone et al. 1965: 612; Weisman 1965: 266, 1966a: 53, 1966b :196; Cole and Schlinger 1969: 331; Telford 1975: 21.

##### Type locality.

Gold Lake Camp, Plumas County, California. [USNM]

##### Diagnosis.

Can be confused with *S.columbiana*, *S.cryptica* sp. n., *S.hoguei* sp. n., *S.oraria* sp. n., and *S.pattonii* but can be distinguished by the following characters: Tergite 1 with uninterrupted, pruinose band along posterior margin. Scutellum mixed black and yellow pilose. Ventral calypter with long yellow pile. Sternites 2 to 4 with a posteromedial, triangular region of non-pruinosity on sternites 2 to 4 that is smaller on ensuing sternites. The species can only be distinguished from *S.hoguei* sp. n. by male genitalia in which the narrowest part of the surstylus is about one-fourth the width of base.

##### Redescription.

**Male.** Body length: 11.2–14.4 mm. Wing length: 9.1–10.6 mm. ***Head.*** Face yellow pruinose with shiny, black, medial vitta extending from oral margin to base of antenna; frons broad, about as long as broad at antenna, two-thirds as broad at vertex as at antenna, bare, with yellow pruinosity along posterior three-fourths; vertex triangular, longer than broad, shiny, with ocellar triangle black pilose; postocular border yellow pruinose; postocular and occipital pile yellow; male narrowly dichoptic; antenna black, black pilose, length of segments roughly in a 3:3:2 ratio.

***Thorax.*** Copper shine; postpronotum, scutum and scutellum yellow pilose, except scutum with black pile posteromedially; postalar callus, proepimeron, posterior anepisternum yellow pilose; posterior katepisternum yellow pilose with broadly separated patches; anterior anepimeron yellow pilose; metasternum yellow pilose; postpronotum, anterior fourth of scutellum, broad posterior margin of anepisternum and dorso-posterior corner of katepisternum yellow pruinose; area between postpronota weakly yellow pruinose, except shiny medially; anepimeron shiny; scutum without pruinose vittae; ventral calypter with long yellow pile.

***Legs.*** Foreleg black, except extreme apex of femur and anterior third of tibia reddish-yellow; fore tarsi slightly broadened; midleg reddish-yellow, except basal four-fifths of femur and last two tarsomeres black; hind leg reddish-yellow, except basal four-fifths of femur and last two tarsomeres black; legs yellow pilose, except fore tibia, fore tarsi, apex of fore femur and last two mid and hind tarsomeres black pilose; hind coxa yellow pruinose.

***Wing.*** Hyaline; microtrichia absent from following areas: cell bc; cell r_1_ from base to about halfway to crossvein r-m; broad basal portion of cell br (before origin of M) and about basal two-fifths of narrower portion of this cell (caudad of spurious vein only); cell bm, except apex and narrow anterior and posterior margins of about apical fourth; broad anterior margin of cell cua; narrow, elongate, oval area proximal to vein A_1_.

***Abdomen.*** Tergites and sternites shiny to sub-shiny, black with yellow pruinose markings as follows: tergite 1 pruinose along posterior margin; tergite 2 with broad, interrupted, truncate, medial band which meets a narrow, uninterrupted, posterior band in the posterolateral corners of tergite; tergite 3 with similar medial band, but more narrowly interrupted; pattern on tergite 4 same as tergite 3 except medial band very narrowly or incompletely interrupted; sternite 1 shiny; sternites 2 to 4 almost completely pruinose, with a triangular region of non-pruinosity posteromedially, with each ensuing region smaller; sternites 6 to 8 pruinose; abdominal pile yellow.

***Male genitalia.*** Surstylus elongate, curving upward dorsally, more than three times as long as broad, about fourth the width of base at narrowest point; pile on dorsal surface of surstylus, symmetric in length; minute spines on ventral surface, with apical four-fifth of lateral inner surface also with spines; basal fourth of the ventral surface of the surstylus not produced into a lobe, but instead with slight invagination and no minute pubescence present; cerci with slight invagination on posterior border; aedeagus as in Fig. 2E.

##### Female.

Similar to male except normal sexual dimorphism.

##### Distribution.

U.S.A.: California, Oregon and Nevada (Fig. 25). Throughout the Sierra Nevada Mountains and Warner Mountains along with the portions of the Cascade Range, Klamath Mountains and the Northern Coast Ranges surrounding the Great Valley.

##### Biology.

Species collected visiting flowers *Ceanothuscuneatus* (Hook.) Nutt. and recorded leafsitting on *Veratrumcalifornicum* Durand. Recorded flying mid-May through mid-August.

#### Sphecomyia hoguei

Taxon classificationAnimaliaDipteraSyrphidae

Moran sp. n.

http://zoobank.org/75E07314-220F-40C8-AD72-A0FA726FED36

Figs 2F , 7B , 8B , 9B , 25

##### Type locality.

U.S.A.: California: San Bernardino County, Summit of Mount Sorenson west of Running Springs, 34.237778, −117.155833, 1912 m.

**Type. *Holotype*** male, pinned. Original label: “USA: CA: San Bernardino Co. // Summit of Mount Sorenson // W of Running Springs; 1912 m // 31°14’16”N, 117°09’21”W // 8.vi.2003; J&A. Skevington” “CNCDiptera // # 110224” “*Sphecomyia // dyari* // [Handwritten] Det. J. Skevington, 2003”, “Leg removed // for DNA // analysis”. [1♂, CNC_Diptera110224, CNC]

***Paratypes*** : U.S.A., CALIFORNIA: Idyllwild, San Jacinto Mountains, 33.7456 -116.7161, J.W. MacSwain, 6.vii.1950, CNC143023 (1♂, CNC); 23.v.1940, CNC143019 (1♂, CNC); Los Angeles Co., Camp Baldy, 34.2689 -117.6286, 1981 m, R. DeNoble, 26.vi.1950, EMEC371314 (1♂, EMEC); R.C. Bechtel, 26.vi.1956, KMM0897 (1♂, CAS); R.W. Bushing, 26.vi.1956, KMM0896 (1♂, CAS); KMM0789 (1♂, SEMC); 7.vii.1958, KMM0898 (1♀, SEMC); San Bernardino Co., Forest Home, 34.0887. −116.9315, E.C. Van Dyke, 17.vi.1928, KMM0895 (1♂, CAS); San Bernardino Co., San Bernardino Mountains, Santa Ana River at Camp Metoche, 34.18, −116.88, 1710 m, J.N. Hogue, 8–9.vi.2013, LACM342617 (1♂, LACM); San Bernardino Co., Snow Crest Camp, 34.2546 -117.6337, A.A. Grigarick, 7.vii.1952, CNC143018 (1♀, CNC); D.E. Barcus, 7.vii.1952, KMM0899 (1♀, SEMC); E.M. Evans, 7.vii.1952, KMM0790 (1♀, SEMC); San Bernardino Co., Up Santa Ana River, 34.1465, −117.0563, J. & G. Sperry, 6.vi.1956, USNM1071433 (1♂, USNM); San Bernardino. Co., San Antonio Falls NE Mount Baldy P.O., 34.2719, −117.6342, *Rhamnuscalifornica* in flower, J. Powell, 18.vi.1981, EMEC371306 (1♀, EMEC).

##### Diagnosis.

It can be confused with *S.columbiana*, *S.cryptica* sp. n., *S.dyari*, *S.oraria* sp. n. and *S.pattonii* but can be distinguished by the following characters: tergite 1 with uninterrupted, pruinose band along posterior margin; scutellum mixed black and yellow pilose; ventral calypter with long yellow pile; sternites 2 to 4 with a posteromedial, triangular region of non-pruinosity on sternites 2 to 4 that is smaller on ensuing sternites. The species can only be distinguished from *S.hoguei* sp. n. by male genitalia in which the narrowest part of the surstylus is about one half the width of base.

##### Description.

**Male.** Body length: 12.5–14.7 mm. Wing length: 8.8–10.5 mm. ***Head.*** Face yellow pruinose with shiny, black, medial vitta extending from oral margin to base of antenna; frons broad, about as long as broad at antenna, two-thirds as broad at vertex as at antenna, bare, with yellow pruinosity along posterior three-fourths; vertex triangular, longer than broad, shiny, with ocellar triangle black pilose; postocular border yellow pruinose; postocular and occipital pile yellow; male narrowly dichoptic; antenna black, black pilose, length of segments roughly in a 3:3:2 ratio.

***Thorax.*** Copper shine; postpronotum, scutum and scutellum yellow pilose, except scutum with black pile posteromedially; postalar callus, proepimeron, posterior anepisternum yellow pilose; posterior katepisternum yellow pilose with broadly separated patches; anterior anepimeron yellow pilose; metasternum yellow pilose; postpronotum, anterior fourth of scutellum, broad posterior margin of anepisternum and dorso-posterior corner of katepisternum yellow pruinose; area between postpronota yellow pruinose, except shiny medially; anepimeron shiny; scutum without pruinose vittae; ventral calypter with long yellow pile.

***Legs.*** Foreleg black, except extreme apex of femur and anterior third of tibia reddish-yellow; fore tarsi slightly broadened; midleg reddish-yellow, except basal four-fifths of femur and last two tarsomeres black; hind leg reddish-yellow, except basal four-fifths of femur and last two tarsomeres black; legs yellow pilose, except fore tibia, fore tarsi, apex of fore femur and last two mid and hind tarsomeres black pilose; hind coxa yellow pruinose.

***Wing.*** Hyaline; microtrichia absent from following areas: cell bc; cell r_1_ from base to about halfway to crossvein r-m; broad basal portion of cell br (before origin of M) and about basal two-fifths of narrower portion of this cell (caudad of spurious vein only); cell bm, except apex and narrow anterior and posterior margins of about apical fourth; broad anterior margin of cell cua; narrow, elongate, oval area proximal to vein A_1_.

***Abdomen.*** Tergites and sternites shiny to sub-shiny, black with yellow pruinose markings as follows: tergite 1 pruinose along posterior margin; tergite 2 with broad, interrupted, truncate medial band which meets a narrow, uninterrupted posterior band in the posterolateral corners of tergite; tergite 3 with similar medial band, but more narrowly interrupted; pattern on tergite 4 same as tergite 3 except medial band very narrowly or incompletely interrupted; sternite 1 shiny; sternites 2 to 4 almost completely pruinose, with a triangular region of non-pruinosity posteromedially, with each ensuing region smaller; sternites 6 to 8 pruinose; abdominal pile yellow.

***Male genitalia.*** Surstylus elongated, curving upward dorsally, more than three times as long as broad, no less than half the width of base at narrowest point; pile on dorsal surface of surstylus, symmetric in length; minute spines on ventral surface, with apical four-fifths of lateral inner surface also with spines; basal fourth of the ventral surface of the surstylus not produced into a lobe, with no invagination or minute pubescence present; cerci with slight invagination on posterior border; aedeagus as in Fig. 2F.

##### Female.

Similar to male except normal sexual dimorphism.

##### Distribution.

U.S.A.: California (Fig. 25). Known from San Gabriel, San Bernardino, and San Jacinto mountains.

##### Biology.

Collected visiting flowers of *Frangulacalifornica* (Eschsch.) A. Gray. Recorded flying late May through early July.

**Etymology.** The specific epithet honors J. N. (Jim) Hogue who collected many of the specimens of *S.hoguei* sp. n., *S.interrupta* sp. n., and *S.sexfasciata* sp. n.

#### Sphecomyia interrupta

Taxon classificationAnimaliaDipteraSyrphidae

Moran sp. n.

http://zoobank.org/8C026307-8B40-49C0-B213-6D5B6223E841

Figs 2G , 4A , 5A , 16C , 17C , 18C , 21F , 22B , 23

##### Type locality.

U.S.A. California: San Bernardino Co., Summit of Heap's Peak west of Running Springs, 34.2347, −117.1397, 1957 m.

**Types. *Holotype*** male, pinned. Original label: “USA: CA: San Bernardino Co. // Summit of Heap’s Peak W. // Of Running Springs; 1957 m // 34°14’05” N, 117°08’23” W // 25.v.2003; J. Skevington”, “CNCDIPTERA // #110220”, “*Sphecomyia // brevicornis* // [Handwritten] Det. J. Skevington, 2003”, “Leg removed // for DNA // analysis”. [1♂, CNC_DIPTERA110220, CNC]

***Paratypes*** : U.S.A., CALIFORNIA: Camp Angelus, 34.1461, −116.9825, white *Ceanothus*, A.L. Melander, 20.v.1947, KMM0900 (1♂, RMNH); San Bernardino Co., Mill Creek, 34.0972, −117.0289, 1828–1920 m, on *Ceanothus*, Timberlake, 30.v.1934, UCRC442807 (1♂, UCRC); San Bernardino Co., San Antonio Canyon, 34.160256, −117.678477, 1889–1950 m, J.N. Hogue, 20.vi.1968, LACM329893 (1♀, LACM).

##### Diagnosis.

Species similar to *S.brevicornis* and *S.sexfasciata* sp. n. but can be distinguished by the following characters: scutum with two pairs of pruinose vittae; cell c completely microtrichose; antenna possessing a 3:3:2 ratio of segments; frons bare; anepimeron not pruinose; scutellum entirely pruinose; medial facial vitta interrupted by a macula of pruinosity on tubercle.

##### Description.

**Male.** Body length: 12.5–14.0 mm. Wing length: 8.9–10.7 mm. ***Head.*** Face yellow pruinose with shiny, black, medial vitta extending from oral margin to base of antenna, except interrupted on facial tubercle by yellow pruinosity; frons broad, about as long as broad at antenna, two-thirds as broad at vertex as at antenna, bare, with yellow pruinosity along posterior half; vertex triangular, longer than broad, shiny, with ocellar triangle black pilose; postocular border yellow pruinose; postocular pile black; occipital pile yellow; male narrowly dichoptic; antenna black, black pilose, with length of segments roughly in a 3:3:2 ratio.

***Thorax.*** Matte black; postpronotum yellow pilose; scutum yellow pilose, except with black pile posteromedially; scutellum, postalar callus, proepimeron, posterior anepisternum yellow pilose; posterior katepisternum yellow pilose with broadly separated patches; anterior anepimeron yellow pilose; metasternum yellow pilose; postpronotum, anterior three-fourths of scutellum, broad posterior margin of anepisternum and dorso-posterior corner of katepisternum yellow pruinose; anepimeron shiny; scutum with two pairs of pruinose vittae: anterior pair long, running from anterior edge of scutum to transverse suture; posterior pair shorter and terminating before posterior edge; ventral calypter with long yellow pile.

***Legs.*** Foreleg reddish-yellow, except basal four-fifths of femur and last two tarsomeres black; midleg reddish-yellow, except basal four-fifths of femur and last two tarsomeres black; hind leg reddish-yellow, except last two tarsomeres black; legs yellow pilose, except black pilose on last three tarsomeres; hind coxa yellow pruinose.

***Wing.*** Hyaline; microtrichia absent from following areas: cell bc; narrow anteromedial region of cell bm; broad anterior margin of cell cua.

***Abdomen.*** Tergites and sternites shiny to sub-shiny, black with yellow pruinose markings as follows: tergite 1 pruinose along posterior margin; tergite 2 with broad, interrupted, truncate medial band which meets a broad, uninterrupted posterior band in the posterolateral corners of tergite; tergite 3 with broad medial band, sometimes very narrowly interrupted, that joins with broad posterior band in two places creating a medial diamond-shaped spot of no pruinosity; pattern on tergite 4 same as tergite 3; sternite 1 pruinose on posterior half; sternites 2 to 4 completely pruinose; sternites 6 to 8 pruinose; pile of abdomen yellow.

***Male genitalia.*** Surstylus elongated, about two and a half times as long as broad, apex acute, with rounded curve, directed ventrally; pile on dorsal surface of surstylus, increasing in length posteriorly; minute spines on ventral surface and apical three-fourth of lateral inner and outer surface; basal fourth of the ventral surface of the surstylus produced into a conspicuous lobe which extends ventrally, with minute pubescence on ventral and lateral inner surface; cerci rounded, with invagination on posterior border; aedeagus as in Fig. 2G.

##### Female.

Medial, facial vittae not interrupted.

##### Distribution.

U.S.A.: California (Fig. 23). Known only from the San Bernardino Mountains.

##### Biology.

Collected visiting flowers of *Ceanothus* L. Recorded flying late May to late June.

**Etymology.** The specific epithet is derived from the Latin *interruptus* (Brown 1956: 441) which means broken apart, between, off, or asunder. It references that the medial facial vitta is interrupted on the tubercle by a macula of pruinosity.

#### Sphecomyia metallica

Taxon classificationAnimaliaDipteraSyrphidae

(Bigot, 1882), stat. rev. and comb. n.

Figs 2H , 4B , 16E , 17E , 18E , 26

Eurhinomallota metallica Bigot 1882: 78. Type Locality: ?California [see below] [UMO]
Brachymyia lupina Williston 1882a: 77. Type Locality: California. **Syn. nov.** [USNM]
Eurhinomallota lupina Williston 1882b: 330.
Criorhina lupina Williston 1886: 209 – Kertész 1910: 288; Curran 1925f: 157; Byers et al. 1962: 167; Nayar 1968: 297; Cole and Schlinger 1969: 330; Telford 1975: 20.

##### Diagnosis.

*Sphecomyiametallica* is not easily confused with any other congeneric as it is the only species which is long pilose and also completely pruinose on the scutum and scutellum.

##### Redescription.

**Male.** Body length: 9.2–13.2 mm. Wing length: 7.9–10.7 mm. ***Head.*** Face silver pruinose with shiny, black, medial vitta extending from oral margin to tubercle; frons broad, about as long as broad at antenna, as broad at vertex as at antenna, pale pilose and silver pruinose; vertex polygonal, slightly longer than broad, silver pruinose, with ocellar triangle pale pilose; postocular border silver; postocular and occipital pile pale; broadly dichoptic in male; antenna black, pale pilose, length of segments roughly in a 3:3:2 ratio.

***Thorax.*** Black; long pilose; postpronotum, scutum, scutellum, postalar callus, proepimeron, posterior anepisternum pale pilose; posterior katepisternum pale pilose with broadly separated patches; anterior anepimeron pale pilose; metasternum pale pilose; postpronotum, mesonotum, broad posterior margin of anepisternum, dorso-posterior corner of katepisternum and anepimeron silver pruinose.

***Legs.*** Foreleg black, except extreme apex of femur and anterior third of tibia reddish-yellow; mid and hind leg similar; tarsi not modified; leg pale pilose; hind coxa silver pruinose.

***Wing.*** Hyaline; wing completely microtrichose.

***Abdomen.*** Tergites and sternites shiny to sub-shiny, black with silver pruinosity as follows: tergite 1 completely silver pruinose; tergite 2 weakly silver pruinose; tergite 3 weakly silver pruinose along margins with thin, interrupted medial band; tergite 4 as tergite 3; sternites 1 to 4 completely silver pruinose; pile of abdomen long, pale.

***Male genitalia.*** Surstylus elongated, about 2½ times as long as broad, apex acute, with rounded curve, directed ventrally; pile on dorsal surface of surstylus, increasing in length posteriorly; minute spines on ventral surface and apical three-fourth of lateral inner and outer surface; basal fourth of the ventral surface of the surstylus produced into a lobe directed ventrally, with minute pubescence on ventral and lateral inner surface; cerci rounded, with conspicuous invagination on posterior border; aedeagus as in Fig. 2H.

##### Female.

Similar to male except normal sexual dimorphism and as follows: medial facial vittae extends past tubercle to terminate just below antenna.

##### Distribution.

U.S.A.: California, Oregon (Fig. 26). Mostly restricted to California, with a short extension into coastal Oregon.

##### Biology.

Associated with lowland *Arctostaphylos* Adans. sp., more commonly known as manzanitas or bearberries. The plant ranges from small shrubs to trees of over 6 m. It has small, clustered, bell-shaped, pink or white flowers. Also collected on flowers of *Ribessanguineum* Pursh and *Ribesmenziesii* Pursh. Due to their unusual flight period of December through mid-April, more research is necessary to reveal the true distribution of the species.

##### Remarks.

Although the type locality is listed as Mexico, the authors believe that the type is from current-day California as it was collected prior to 1848 when the state was still part of Mexico.

Contrary to the previous treatment, *Eurhinomallotametallica* Bigot, 1882 is senior to *Brachymyialupina* Williston, 1882. Bigot’s name was published in the bimonthly Bulletin de la Société entomologique de France in March of 1882. Williston’s name was published in the April 1882 issue of the Canadian Entomologist. The improper treatment arose because the Bulletin itself was obscure until recently, with the Annales de la Société entomologique de France, the annually published compilation, taken as the date of publication for many species.

The combination of *Eurhinomallota* with *Sphecomyia* is supported by the type species’ possession of all characters used to distinguish *Sphecomyia* from other Criorhinina. This decision is further supported by molecular evidence showing a close relationship with *Sphecomyia*, i.e., the present COI gene tree (Fig. 27) and a multi-gene molecular phylogeny of the Criorhinina which will be presented in an upcoming paper. It is the authors opinion that combination with *Sphecomyia*, as opposed to resurrecting the concept as a monotypic genus, serves to emphasize its relationship with the group.

#### Sphecomyia oraria

Taxon classificationAnimaliaDipteraSyrphidae

Moran sp. n.

http://zoobank.org/36910837-6671-464C-B22A-5C0D95EAEDAC

Figs 2I , 7E , 8E , 9E , 15B , 20A , 21C , 25

##### Type locality.

U.S.A.: California: Marin County, 2 miles SE Inverness, Inverness Ridge, 38.1014, −122.8869.

##### Types.

***Holotype*** male, pinned. Original label: “CALIF: Marin Co., // 2 mi SE Inverness, // Inverness Ridge, // at light, May 15, // 1970, J. A. Powell” “Univ. Calif. // Insect Survey // Specimen # // 111082” “UC Berkley // EMEC // 371304 // [BARCODE]”. [1♂, EMEC371304, EMEC].

***Paratypes*** : U.S.A.: California: Humboldt Co., Blocksburg, 40.2756 -123.6364, B.P. Bliven, 30.v.1937, KMM0894 (1♀, CAS); Marin Co., 2 mi. SE Inverness, Inverness Ridge, 38.1014, −122.8870, 243–316 m, H. Ewing, 7.v.1971, EMEC371305 (1♂, EMEC); Marin Co., Lily Pond, Alpine Lake, 37.9538, −122.6349, 457 m, D.D. Munroe, 10.v–4.vi.1970, CNC47070 (1♀, CNC); Mendocino Co., NCCRP, 3 mi. N. Branscomb, 39.6464 -123.4470, 427 m, C. Strong, 21–23.v.1982, EMEC371315 (1♀, EMEC); San Luis Obispo Co., Atascadero, 35.4883 -120.6703, J. LeCroy, 4.v.1986, LACM329903 (1♀, LACM); Santa Clara Co., Creek along Sandborn road, 2.7 km SE Congree-Springs road, 37.2347, −122.0589, 440 m, P.H. Arnaud, Jr., 14.iv.1974, USNM1028896 (1♂, USNM); Sonoma Co., Plantation, 38.5903 -123.3103, D. Burdick, 1.v.1958, INHS776993 (1♂, INHS); Sonoma Co., Stillwater Cove, 38.5424, −123.2888, E.I. Schlinger, 23.v.1954, KMM0893 (1♂, CAS); USNM1028841 (1♂, CNC); Walnut Creek, 37.9103, −122.0653, v, USNM1028837 (1♀, USNM).

##### Diagnosis.

It can be confused with *S.columbiana*, *S.cryptica* sp. n., *S.dyari*, *S.hoguei* sp. n., and *S.pattonii* but can be distinguished by the following characters: tergite 1 with uninterrupted, pruinose band along posterior margin. Scutellum black pilose. Ventral calypter with long yellow pile. Sternites 2 to 4 mostly pruinose, with narrow anterior border and transverse subapical band shiny to dull black.

##### Description.

**Male.** Body length: 11.1–14.6 mm. Wing length: 8.9–11.6 mm. ***Head.*** Face yellow pruinose with shiny, black, medial vitta extending from oral margin to base of antenna; frons broad, about as long as broad at antenna, two-thirds as broad at vertex as at antenna, bare, with yellow pruinosity along posterior half; vertex triangular, longer than broad, shiny, with ocellar triangle black pilose; postocular border yellow pruinose; postocular pile black; occipital pile yellow; male narrowly dichoptic; antenna black, black pilose, with length of segments roughly in a 3:3:2 ratio.

***Thorax.*** Sub-shiny black; postpronotum yellow pilose with occasional black pile; scutum, scutellum and postalar callus mostly black pilose with occasional yellow pile; proepimeron and posterior anepisternum yellow pilose; posterior katepisternum yellow pilose with broadly separated patches; anterior anepimeron yellow pilose; metasternum yellow pilose; postpronotum, anterior fourth of scutellum, broad posterior margin of anepisternum and dorso-posterior corner of katepisternum yellow pruinose; area between postpronota yellow pruinose, except shiny medially; anepimeron shiny; scutum without pruinose vittae; ventral calypter with long yellow pile.

***Legs.*** Foreleg black, except extreme apex of femur and anterior third of tibia reddish-yellow; fore tarsi slightly broadened; midleg reddish-yellow, except basal four-fifths of femur and last two tarsomeres black; hind leg reddish-yellow except last two tarsomeres black; legs yellow pilose, except fore tibia, fore tarsi, apex of fore femur and last two mid and hind tarsomeres black pilose; hind coxa yellow pruinose.

***Wing.*** Hyaline; microtrichia absent from following areas: cell bc; cell r_1_ from base to about halfway to crossvein r-m; broad basal portion of cell br (before origin of M) and about basal two-fifths of narrower portion of this cell (caudad of spurious vein only); cell bm, except apex and narrow anterior and posterior margins of about apical fourth; broad anterior margin of cell cua; narrow, elongate, oval area proximal to vein A_1_.

***Abdomen.*** Tergites and sternites shiny to sub-shiny, black with yellow pruinose markings as follows: tergite 1 pruinose along posterior margin; tergite 2 with broad, interrupted, narrowing, medial band which meets a narrow, uninterrupted posterior band in the posterolateral corners of tergite; tergite 3 with similar, but truncate, medial band, but more narrowly interrupted; pattern on tergite 4 same as tergite 3 except medial band very narrowly or incompletely interrupted; sternite 1 shiny; sternites 2 to 4 mostly pruinose, each with narrow anterior border and transverse subapical band shiny to dull black; sternites 6 to 8 pruinose; pile of abdomen yellow, except sometimes with scattered black pile present on postabdomen.

***Male genitalia.*** Surstylus not elongated, about as long as broad, curving upward dorsally; pile on dorsal surface of surstylus, increasing in length posteriorly; minute spines on ventral surface, with apical half of lateral inner surface also with spines; basal fourth of the ventral surface of the surstylus produced into a lobe directed anteriorly, with minute pubescence on ventral and lateral inner surface; cerci rounded, with no invagination on posterior border; aedeagus as in Fig. 2I.

##### Female.

Similar to male except normal sexual dimorphism.

##### Distribution.

U.S.A.: California (Fig. 25). A lowland species spread throughout the California Coast Ranges.

##### Biology.

Recorded flying late April through May.

**Etymology.** The specific epithet is derived from the Latin *orarius* (Brown 1956: 576), meaning ‘of the coast’.

#### Sphecomyia pattonii

Taxon classificationAnimaliaDipteraSyrphidae

Williston, 1882

Figs 2J , 7F , 8F , 9F , 11B , 20B , 25

Sphecomyia pattonii Williston 1882b: 328 – Kertész 1910: 349; Vockeroth 1965: 86; Stone et al. 1965: 613; Weisman 1965: 268, 1966a: 53, 1966b :194; Boyes and van Brink 1967: 432, 1970: 212; Cole and Schlinger 1969: 331; Telford 1975: 21; Hippa 1978: 15. **Type locality.** “Washington Territory”. [USNM]
Calliprobola calorhina Bigot 1884: 353 – Williston 1887: 258. **Type locality.** “Washington Territory”. [UMO]
Sphecomyia pattoni Williston 1886: 258 – Aldrich 1905: 404; Osburn 1908: 14; Shannon 1925: 43; Curran 1932: 8.

##### Diagnosis.

Species similar to S.columbiana, S.cryptica sp. n., S.dyari, S.hoguei sp. n. and S.oraria sp. n. but can be distinguished by the following characters: tergite 1 with uninterrupted, pruinose band along posterior margin; scutellum black pilose; ventral calypter with long black pile; sternites 2 to 4 mostly pruinose, with narrow anterior border and transverse subapical band shiny to dull black.

##### Redescription.

**Male.** Body length: 12.1–16.0 mm. Wing length: 8.3–11.8 mm. ***Head.*** Face yellow pruinose with shiny, black, medial vitta extending from oral margin to base of antenna; frons broad, about as long as broad at antenna, two-thirds as broad at vertex as at antenna, bare, with yellow pruinosity along posterior fourth; vertex triangular, longer than broad, shiny, with ocellar triangle black pilose; postocular border yellow pruinose; postocular pile black, occipital pile yellow; male narrowly dichoptic; antenna black, black pilose, with length of segments roughly in a 3:3:2 ratio.

***Thorax.*** Sub-shiny black; postpronotum yellow pilose with occasional black pile; scutum and scutellum mostly black pilose with occasional yellow pile; postalar callus mixed black and yellow pilose; proepimeron, posterior anepisternum yellow pilose; posterior katepisternum yellow pilose with broadly separated patches; anterior anepimeron yellow pilose; metasternum yellow pilose; postpronotum, anterior fourth of scutellum, broad posterior margin of anepisternum and dorso-posterior corner of katepisternum yellow pruinose; area between postpronota yellow pruinose, except shiny medially; anepimeron shiny; scutum without pruinose vittae; ventral calypter with long black pile.

***Legs.*** Foreleg black, except extreme apex of femur and anterior third of tibia reddish-yellow; fore tarsi slightly broadened; midleg reddish-yellow, except basal four-fifths of femur and last two tarsomeres black; hind leg reddish-yellow except last two tarsomeres black; legs yellow pilose, except fore tibia, fore tarsi, apex of fore femur and last two mid and hind tarsomeres black pilose; hind coxa yellow pruinose.

***Wing.*** Hyaline; microtrichia absent from following areas: cell bc; cell r_1_ from base almost to crossvein r-m; broad basal portion of cell br (before origin of M) and about basal two-fifths of narrower portion of this cell (caudad of spurious vein only); cell bm except apex and narrow anterior and posterior margins of about apical fourth; broad anterior margin of cell cua; narrow, elongate, oval area proximal to vein A_1_.

***Abdomen.*** Tergites and sternites shiny to sub-shiny, black with yellow pruinose markings as follows: tergite 1 pruinose along posterior margin; tergite 2 with broad, interrupted, narrowing medial band which meets a narrow, uninterrupted posterior band in the posterolateral corners of tergite; tergite 3 with similar, but truncate, medial band more narrowly interrupted; pattern on tergite 4 same as tergite 3 except medial band very narrowly or incompletely interrupted; sternite 1 shiny; sternites 2 to 4 mostly pruinose, each with narrow anterior border and transverse subapical band shiny to dull black; sternites 6 to 8 pruinose; pile of abdomen yellow, except sometimes with scattered black pile present on postabdomen.

***Male genitalia.*** Surstylus elongated, about 1½ times as long as broad, curving upward dorsally; pile on dorsal surface of surstylus, increasing in length posteriorly; minute spines on ventral surface of surstylus, with apical three-fourths of lateral inner surface also with spines; basal fourth of the ventral surface of the surstylus produced into a lobe directed anteriorly, with minute pubescence on ventral and lateral inner surface; cerci rounded, with no invagination on posterior border; aedeagus as in Fig. 2J.

##### Female.

Similar to male except normal sexual dimorphism.

##### Distribution.

U.S.A.: Washington, Oregon, Idaho, Montana. Canada: British Columbia (Fig. 25). Widespread throughout coastal and mountainous areas of Washington state except seemingly absent from the Columbia basin. Extends into coastal and forested parts of northeastern Oregon. Extends north into forested coastal and inland areas of British Columbia. Also known from forests of northern Idaho and western Montana.

##### Biology.

The authors collected this species visiting Rubus L. sp. on a forested slope near a river. Also collected visiting flowers of Heracleumlanatum Michx. Known hilltopper. Recorded flying late April through mid-August, with one outlier from mid-October.

#### Sphecomyia pseudosphecomima

Taxon classificationAnimaliaDipteraSyrphidae

Moran sp. n.

http://zoobank.org/3371F4A6-3010-416D-9260-EA89EB01DE69

Figs 10C , 12C , 26

##### Type locality.

U.S.A.: California: Tulure Co., Ash Mountain Headquarters, 36.4868, −118.8398, 518 m.

##### Types.

***Holotype*** female, pinned. Original label: CAL: Tulare Co. // Ash Mt. HQ, 1700’ // IV-28-1979 // J. Powell, coll.” “EMEC // 371308 // [BARCODE]”. [1♀, EMEC371308, EMEC]

***Paratypes*** : U.S.A.: California: Kern Co., Glennville, 35.7236, −118.7021, E.G. Linsley, J.W. MacSwain, R.F. Smith, 24.iv.1949, CNC91444 (1♀, CNC); Yosemite National Park, 37.7399, −119.5911, E.C. Van Dyke, 16.v.1921, USNM1028990 (1♀, USNM).

##### Diagnosis.

Species similar to *S.aino* or *S.tsherepanovi* but can be distinguished by the following characters: cell c bare on basal third; ocellar triangle pale pilose; silver-yellow pruinose.

##### Description.

**Female.** Body length: 9.9 –12.7 mm. Wing length: 7.7–7.9 mm. ***Head.*** Face silver-yellow pruinose with shiny, black, medial vitta extending from oral margin to base of antenna; frons black pilose posteriorly, silver-yellow pruinose on lateral margins; postocular border silver-yellow pruinose; postocular and occipital pile pale; antenna black, black pilose, with length of segments roughly in a 3:3:2 ratio.

***Thorax.*** Sub-shiny black; postpronotum, scutum, scutellum, postalar callus, proepimeron, posterior anepisternum pale pilose; posterior katepisternum pale pilose with broadly separated patches; anterior anepimeron pale pilose; metasternum pale pilose; postpronotum, anterior eighth of scutellum, broad posterior margin of anepisternum and dorso-posterior corner of katepisternum silver-yellow pruinose; area between postpronota weakly silver-yellow pruinose, except shiny medially; anepimeron shiny; scutum without pruinose vittae; ventral calypter with long yellow pile.

***Legs.*** Foreleg black except extreme apex of femur; midleg reddish-yellow, except last two tarsomeres black; hind leg reddish-yellow except last two tarsomeres black; all of fore tibia and tarsus black pilose, remainder of leg pale pilose.

***Wing.*** Hyaline; microtrichia absent from following areas: cell bc; basal third of cell c; basal fourth of cell sc; cell r_1_ from base almost to crossvein r-m; broad basal portion of cell br (before origin of M) and about basal two-fifths of narrower portion of this cell (caudad of spurious vein only); cell bm except apex and narrow anterior and posterior margins of about apical fourth; broad anterior margin of cell cua; narrow, elongate, oval area proximal to vein A_1_.

***Abdomen.*** Tergites and sternites shiny to sub-shiny, black with silver-yellow pruinosity as follows: tergite 1 pruinose posteriorly; tergite 2 with thin, interrupted, medial band which curves posteriorly to reach the posterolateral corners; tergite 3 with thin, interrupted, medial band which does not curve anteriorly; tergite 4 with similar but thinner band; sternite 1 shiny; sternites 2 to 4 pruinose, with indistinct spot of non-pruinosity posteromedially; pile of abdomen pale.

##### Male.

Unknown.

##### Distribution.

U.S.A.: California (Fig. 26). Known from three localities in the Sierra Nevada Range.

##### Biology.

Recorded flying late April through mid-May.

**Etymology.** The specific epithet is derived from the Greek *pseudo* (Brown 1956: 652) meaning false and *sphex* meaning wasp (Brown 1956: 652) and the latin *mima* (Brown 1956: 652) for mimic. The epithet referencing that it is one of the few non-wasp mimics of *Sphecomyia*.

#### Sphecomyia sexfasciata

Taxon classificationAnimaliaDipteraSyrphidae

Moran sp. n.

http://zoobank.org/B7776D23-4486-45D1-AB1E-A34A0CDD75C4

Figs 2K , 16B , 17B , 18B , 23

##### Type locality.

U.S.A: California, Ventura Co., Ventura Mountains, Pine Mountain Creek, just south of Reyes Creek Campground, 34.677, −119.308, 1190 m.

##### Type.

***Holotype*** male, pinned. Original label: “USA: California: Ventura Co. // Ventura Mountains, Pine Mountain // creek just S. of Reyes Cr. Cmpgrd. // 34.677° N, −119.308° W, elev 1190 m // at Prunusvirginiana var. demisa // 29 April–1 May 2016 // J. N. Hogue, notes JNH# 526” “LACM ENT 342251”. [1♂, LACMENT342251, LACM]

***Paratypes*** : U.S.A.: California, Arroyo Seco, 34.118483, −118.191733, C.D. Michener, 27.i.1935, CNC46969 (1♂, CNC); Monterey Co., Highway. 1, roadside canyon 3.5 km N Lucia, 36.0589, −121.5875, K.C. Holston, 15.v.2001, KMM0901 (1♀, CSCA); Riverside Co., Morongo Valley, 34.0451, −116.5668, W. Laidlaw, 28.iv.1972, JSS45129 (1♀, CAS); Riverside Co., Riverside, 33.9533, −117.3919, *Salixlasiolepis*, 26.ii.1933, UCRC428629; UCRC428631 (1♂,1♀, UCRC); San Bernardino Co., Big Morongo Canyon Preserve, 34.0507, −116.5694, J.H. Skevington, K. Moran, 25.iv.2016, CNC517072 (1♀, CNC); Ventura Co., Ventura Mountains, Pine Mountain Creek, just South of Reyes Creek Campground, 34.677, −119.308, 1190 m, Prunusvirginianavar.demisa, A.M. Haberkern, 30.iv.2016, LACMENT342306 (1♂, USNM); J.N. Hogue, 29.iv–1.v.2016, LACMENT342252 (1♂, LACM).

##### Diagnosis.

Species similar to *S.brevicornis* and *S.interrupta* sp. n. but can be distinguished by the following characters: scutum with three pairs of pruinose vittae; cell c completely microtrichose; antenna possessing a 3:3:2 ratio of segments; frons pilose; anepimeron pruinose; anterior three-fourth of scutellum pruinose; medial facial vitta not interrupted by a macula of pruinosity on tubercle.

##### Description.

**Male.** Body length: 12.3–12.6 mm. Wing length: 8.9–9.6 mm. ***Head.*** Face yellow pruinose with shiny, black, medial vitta extending from oral margin to base of antenna; frons broad, about as long as broad at antenna, two-thirds as broad at vertex as at antenna, sparsely yellow pilose, with yellow pruinosity along posterior fourth; vertex triangular, longer than broad, shiny, with ocellar triangle black pilose; postocular border yellow pruinose; postocular pile black; occipital pile yellow; male narrowly dichoptic; antenna black, black pilose, length of segments roughly in a 3:3:2 ratio.

***Thorax.*** Matte black; postpronotum yellow pilose; scutum yellow pilose, except with black pile posteromedially; scutellum, postalar callus, proepimeron, posterior anepisternum yellow pilose; posterior katepisternum yellow pilose with broadly separated patches; anterior anepimeron yellow pilose; metasternum yellow pilose; postpronotum, scutellum, broad posterior margin of anepisternum and dorso-posterior corner of katepisternum yellow pruinose; anepimeron yellow pruinose anteriorly; scutum with three pairs of pruinose vittae, anterior pair long running from anterior edge of scutum to transverse suture, posterior pair shorter and terminating before posterior edge and a small medial pair along the lateral margins of the scutum; ventral calypter with long yellow pile.

***Legs.*** Fore femur, except for extreme apex, along with last two tarsi black; rest of leg yellow; midleg with femur except extreme apex, and last two tarsomeres, black; rest of leg reddish-yellow; hind leg reddish-yellow except last two tarsomeres black; legs yellow pilose, except black pilose on last three tarsomeres;

***Wing.*** Hyaline; microtrichia absent from following areas: broad anterior margin of cell cua.

***Abdomen.*** Tergites and sternites shiny to sub-shiny, black with yellow pruinose markings as follows: tergite 1 pruinose along posterior margin; tergite 2 with broad, interrupted, truncate medial band which meets a broad, uninterrupted posterior band in the posterolateral corners of tergite; tergite 3 with broad medial band, sometimes very narrowly interrupted, that joins with broad posterior band in two places creating a medial diamond-shaped spot of no pruinosity; pattern on tergite 4 same as tergite 3; sternites 1 to 4 completely pruinose; sternites 6 to 8 pruinose; pile of abdomen yellow.

***Male genitalia.*** Surstylus elongated, about 2½ times as long as broad, apex acute, directed ventrally, with abrupt curve; pile on dorsal surface of surstylus, increasing in length posteriorly; minute spines on ventral surface and apical three-fourth of lateral inner and outer surface; basal fourth of the ventral surface of the surstylus produced into a lobe directed ventrally, with minute pubescence on ventral and lateral inner surface; cerci rounded, with invagination on posterior border; aedeagus as in Fig. 2K.

##### Female.

Similar to male except normal sexual dimorphism.

##### Distribution.

U.S.A.: California (Fig. 23). Lowland chaparral in southern California.

##### Biology.

Collected visiting flowers of *Salixlasiolepis* Benth. and Prunusvirginianavar.demisa (Nutt. ex Torr. and A. Gray) Torr. Recorded flying late January through mid-May.

**Etymology.** The specific epithet is derived from the Latin *sex* (Brown 1956: 700), which means six, and the Latin *fasciata* (Brown 1956: 134), which means band or stripe. It references the three pairs of vittae on the scutum, a character unique within the genus *Sphecomyia*.

#### Sphecomyia tsherepanovi

Taxon classificationAnimaliaDipteraSyrphidae

(Violovitsh, 1974), stat. rev. et comb. n.

Figs 2L , 10D , 12D , 13B , 14C , 24

Criorrhina tsherepanovi Violovitsh 1974:127. **Type locality.** Russia: Kuril Islands, Island Sikotan. [ZISP]
Criorhina tsherepanovi Violovitsh 1976:341 – 1982: 211, 1983: 137; Peck 1988:207
Criorrhina aino Mutin and Barkalov 1990:118, not Stackelberg 1955. Misidentification
Criorhina aino of authors, not Stackelberg 1955 – Mutin and Barkalov 1997: 217; Ohishi et al. 2004: 27; Mutin 2016: 17. Misidentification.

##### Diagnosis.

Species similar to S.aino or S.pseudosphecomima but can be distinguished by the following characters: cell c bare on basal two-thirds; ocellar triangle black pilose; silver-white pruinose; basiphallus as in Fig. 2L.

##### Redescription.

**Male.** Body length: 10.9–14.2 mm. Wing length: 8.4–9.0 mm. ***Head.*** Face silver-white pruinose with shiny, black, medial vitta extending from oral margin to base of antenna; frons broad, about as long as broad at antenna, two-thirds as broad at vertex as at antenna, bare, with silver-white pruinosity along posterior rim; vertex triangular, longer than broad, shiny, with ocellar triangle entirely, or at least mostly, black pilose; postocular border silver-white pruinose; postocular pile black; occipital pile pale; male narrowly dichoptic; antenna black, mostly black pilose, with length of segments roughly in a 3:3:2 ratio.

***Thorax.*** Sub-shiny black; postpronotum pale pilose; scutum pale pilose, except with black pile posteromedially; scutellum, postalar callus, proepimeron, posterior anepisternum pale pilose; posterior katepisternum pale pilose with broadly separated patches; anterior anepimeron pale pilose; metasternum pale pilose; postpronotum, anterior eighth of scutellum, broad posterior margin of anepisternum and dorso-posterior corner of katepisternum silver-white pruinose; area between postpronota weakly silver-white pruinose, except shiny medially; anepimeron shiny; scutum without pruinose vittae; ventral calypter with long pale pile.

***Legs.*** Foreleg black, except reddish-yellow at apex of femur; fore tarsi slightly broadened; midleg yellow, except basal four-fifths of femur and last two tarsomeres black; hind leg reddish-yellow, except last two tarsomeres black; legs pale pilose, except black pilose on fore tibia, fore tarsi, extreme apex of fore femur and last two mid and hind tarsomeres; hind coxa silver-white pruinose.

***Wing.*** Hyaline; microtrichia absent from following areas: cell bc; basal two-thirds of cell c; basal fourth of cell sc; cell r_1_ from base almost to crossvein r-m; broad basal portion of cell br (before origin of M) and about basal two-fifths of narrower portion of this cell (caudad of spurious vein only); cell bm, except apex and narrow anterior and posterior margins of about apical fourth; broad anterior margin of cell cua; narrow, elongate, oval area proximal to vein A_1_.

***Abdomen.*** Tergites and sternites shiny to sub-shiny, black with silver-white pruinosity as follows: tergite 1 pruinose posteriorly; tergite 2 with thin, interrupted, medial band which curves posteriorly to reach the posterolateral corners; tergite 3 with thin, interrupted, medial band which does not curve anteriorly; tergite 4 with similar but thinner band; sternite 1 weakly pruinose; sternites 2 and 3 pruinose on anterior third and sub-shiny on remainder; sternite 4 with anteromedial pruinose spots; pile of abdomen pale.

***Male genitalia.*** Surstylus not elongated, about as long as broad, curving upward ventrally; pile on anterolateral outer surface of surstylus; minute spines on ventral surface and apical half of interior lateral surface; basal fourth of the ventral surface of the surstylus produced into a lobe directed anteriorly, with minute pubescence on ventral and lateral inner surface; cerci rounded, with no invagination on posterior border; aedeagus as in Fig. 2L.

##### Female.

Similar to male except normal sexual dimorphism.

##### Distribution.

Japan: Hokkaido, Honshu. Russia: Kuril Islands (Fig. 24).

##### Biology.

Collected visiting flowers of Philadelphussatsumi Siebold ex Lindl. and J. Paxton. Recorded flying early June through mid-July.

##### Remarks.

See S.aino.

#### Sphecomyia vespiformis

Taxon classificationAnimaliaDipteraSyrphidae

(Gorski, 1852)

Figs 2M , 4C , 16D , 17D , 18D , 19B , 21E , 22F , 24

Tyzenhauzia vespiformis Gorski 1852: 170. **Type locality.** Vilinius, Lithuania. [ZMHU]
Sphecomyia vespiformis , Wahlberg 1854: 155 – Zetterstedt 1855: 4646; 1859: 5075; Schiner 1857: 445, 1862: 367, 1864: 112; Bonsdorff 1861: 213; Siebke 1877: 50; Curran 1932: 8; Bańkowska 1963: 67; Stone et al. 1965: 612; Weisman 1965: 268, 1966a: 51, 1966b: 192; Violovitsh 1983: 146; Peck 1988: 213; Soszyński 1991: 92, 2004: 307; Bartsch et al. 1998: 53; Nielsen 1999: 10,91; Söderman 1999: 33; Haarto and Kerppola 2007: 488, 2014: 247; Karpa 2008: 17; Bartsch et al. 2009: 379; Speight 2014: 246; Pettersson and Fors 2014: 6; Mutin et al. 2016: 9; Żóralski et al. 2016: 127, 2017: 76.
Sphecomyia vittata of authors, not Wiedemann 1830 – Osten Sacken 1877: 341; Roder 1879: 96; Portschinsky 1887: 8; Aldrich 1905: 405; Kertész 1910: 349; Shannon 1925: 43; Stackelberg 1958: 244; Séguy 1961: 156; Cole and Schlinger 1969: 331; Peck 1988: 213. Misidentification.

##### Diagnosis.

It can be confused with S.vittata but can be distinguished by the following characters: anepimeron not pruinose; anterior half of scutellum pruinose; sternite 2 completely black or with faint, interrupted, pruinose band anteriorly.

##### Redescription.

**Male.** Body length: 14.8–15.9 mm. Wing length: 10.4–12.1 mm. ***Head.*** Face yellow pruinose with shiny, black, medial vitta extending from oral margin to base of antenna; frons enlarged antero-dorsally, longer than broad and as broad at vertex as at antenna, bare, with yellow pruinosity along posterior rim; vertex triangular, longer than broad, shiny, with ocellar triangle yellow pilose; postocular border yellow pruinose; postocular and occipital pile yellow; male narrowly dichoptic; antenna black, black pilose, with length of segments roughly in a 4:4:1 ratio.

***Thorax.*** Matte black; postpronotum, scutum, scutellum, postalar callus, proepimeron, posterior anepisternum yellow pilose; posterior katepisternum yellow pilose with broadly separated patches; anterior anepimeron yellow pilose; metasternum yellow pilose; postpronotum, anterior half of scutellum, broad posterior margin of anepisternum and dorso-posterior corner of katepisternum yellow pruinose; anepimeron shiny; scutum with two pairs of pruinose vittae, anterior pair long running from anterior edge of scutum to transverse suture, posterior pair shorter and terminating before posterior edge; ventral calypter with long yellow pile.

***Legs.*** Legs yellow to reddish-yellow. Legs yellow pilose.

***Wing.*** Hyaline; microtrichia absent from following areas: cell bc; broad basal portion of cell br (before origin of M) and about basal two-fifths of narrower portion of this cell (caudad of spurious vein only); cell bm anteromedially; broad anterior margin of cell cua.

***Abdomen.*** Tergites and sternites shiny to sub-shiny, black with yellow pruinose markings as follows: tergite 1 pruinose along posterior margin; tergite 2 with broad, interrupted, narrowing medial band which does not meet a narrow, uninterrupted posterior band in the posterolateral corners of tergite; tergite 3 with similar band, but thinner and more narrowly interrupted; pattern on tergite 4 same as tergite 3 except medial band very narrowly or incompletely interrupted; sternite 1 shiny; sternite 2 completely black or with faint, interrupted band anteriorly; sternite 3 and 4 with uninterrupted, or narrowly interrupted band anteriorly; sternites 6 to 8 pruinose; pile of abdomen yellow.

***Male genitalia.*** Surstylus elongated, about two and a half times as long as broad, apex cute, with abrupt curve, directed ventrally; pile on dorsal surface of surstylus, increasing in length posteriorly; minute spines on ventral surface and apical three-fourth of lateral inner and outer surface; basal fourth of the ventral surface of the surstylus produced into a lobe directed posteroventrally, with minute pubescence on ventral and lateral inner surface; cerci rounded, with invagination on posterior border; aedeagus as in Fig. 2M.

##### Female.

Similar to male except normal sexual dimorphism.

##### Biology.

Often found in June or July along rivers and streams in Betula L./Pinus L. forest. Copulation has been observed on the trunk of Populustremula L., Crataegusmaximowiczii C.K.Schneid., Hesperismatronalis L., Pimpinellasaxifraga L., Rubusidaeus L., Sorbusaucuparia L., and Spiraeasalicifolia L. Immature stages are not described but are probably associated with sap-runs or lesions in the trunk of Populustremula (Speight 2014).

##### Distribution.

Southern Norway to northern Sweden, Finland and Russian Karelia, the Baltic States, Poland, and throughout Siberia, reaching the Pacific coast (Fig. 24).

#### Sphecomyia vittata

Taxon classificationAnimaliaDipteraSyrphidae

(Wiedemann, 1830)

Figs 1 , 2N , 5C , 6B , 16F , 17F , 18F , 19A , 21D , 23

Chrysotoxum vittatum Wiedemann 1830: 87. **Type locality.** Unknown. LT male designated in Thompson 1988: 222 [NMW]
Psarus ornatus Wiedemann 1830: 91 – Macquart 1835: 491. **Type locality.** U.S.A.: Georgia [ZMHU]
Sphecomyia vittata , Macquart 1842: 75 – Gorski 1852: 170; Zetterstedt 1855: 4646; Osten Sacken 1875: 62, 1877: 342; Roder 1879: 96; Williston 1886: 257; Portschinsky 1887: 8; Smith 1890: 388; Hunter 1896: 101; Johnson 1900: 664, 1910: 349, 1914: 125, 1925: 178, 1929: 374; Chagnon 1901: 71; Aldrich 1905: 405; Jones 1907: 99; Osburn 1908: 14; Kertész 1910: 349; Metcalf 1913: 98, 1916: 111; Winn and Beaulieu 1915: 138; Banks et al. 1916: 192; Cockerell 1917: 16; Britton 1920: 188; Wehr 1924: 42; Shannon 1925: 43; Leonard 1928: 802; Curran 1932: 8; Winn and Maltais 1932: 53; Brimley 1938: 355; Stone (et al.) 1965: 613; Weisman 1965: 268, 1966a: 50, 1966b: 191; Cole and Schlinger 1969: 331; Waldbauer 1970: 45, 1983: 81; Shorter and Drew 1976: 89; Finnamore and Neary 1978: 172; Maier and Waldbauer 1979: 60; Waldbauer and LaBerge 1985: 101; Thompson 1988: 222.
Sphecomyia boscii Desmarest 1848: 730 – Evenhuis and Thompson 1990: 254. Type locality. U.S.A.: Carolinas. [MNHN]. Syn. n.

##### Diagnosis.

It can be confused with S.vespiformis but can be distinguished by the following characters: anepimeron pruinose; anterior three-fourths of scutellum pruinose; sternite 2 with anterior corners and lateral margins pruinose.

##### Redescription.

**Male.** Body length: 10.9–17.1 mm. Wing length: 7.9–12.1 mm. ***Head.*** Face yellow pruinose with shiny, black, medial vitta extending from oral margin to base of antenna; frons not enlarged antero-dorsally, longer than broad and as broad at vertex as at antenna, bare, with yellow pruinosity along posterior fourth; vertex triangular, longer than broad, shiny, with ocullar triangle yellow, black or mixed black and yellow pilose; postocular border yellow pruinose; postocular and occipital pile yellow; male narrowly dichoptic; antenna black, black pilose, with length of segments roughly in a 4:4:1 ratio.

***Thorax.*** Matte black; postpronotum, scutum completely yellow pilose, except sometimes with black pile posteromedially; scutellum yellow pilose, except sometimes with black pile on non-pruinose portion; postalar callus, proepimeron, posterior anepisternum yellow pilose; posterior katepisternum yellow pilose with broadly separated patches; anterior anepimeron yellow pilose; metasternum yellow pilose; postpronotum, anterior three-fourths of scutellum, broad posterior margin of anepisternum, dorso-posterior corner of katepisternum and yellow pruinose; anepimeron pruinose anteriorly; scutum with two pairs of tear shaped pruinose vittae, anterior pair short stopping before transverse suture, posterior pair longer but terminating before posterior edge; ventral calypter with long yellow pile.

***Legs.*** Legs yellow to reddish-yellow. Legs yellow pilose.

***Wing.*** Hyaline; microtrichia absent from following areas: cell bc; basal third of cell sc; broad basal portion of cell br (before origin of M) and about basal two-fifths of narrower portion of this cell (caudad of spurious vein only); cell bm except apex and narrow posterior margins of about apical half; broad anterior margin of cell cua.

***Abdomen.*** Tergites and sternites shiny to sub-shiny, black with yellow pruinose markings as follows: tergite 1 pruinose along posterior margin; tergite 2 with broad, interrupted, truncate medial band which meets a broad, uninterrupted, posterior band in the posterolateral corners of tergite; tergite 3 with similar band, but more narrowly interrupted; pattern on tergite 4 same as tergite 3 except medial band very narrowly or incompletely interrupted; sternite 1 shiny; sternite 2 with anterior corners and lateral margins pruinose; sternite 3 mostly pruinose with posteromedial region of non-pruinosity, sternite 4 pruinose on anterior third and lateral margins; sternites 6 to 8 pruinose; pile of abdomen yellow.

***Male genitalia.*** Surstylus elongated, about 2½ times as long as broad, apex rounded, directed ventrally; pile on dorsal surface of surstylus, increasing in length posteriorly; minute spines on ventral surface and apical three-fourth of lateral inner and outer surface; basal fourth of the ventral surface of the surstylus produced into a lobe directed posteroventrally, with minute pubescence on ventral and lateral inner surface; cerci rounded, with invagination on posterior border; aedeagus as in Fig. 2N.

##### Female.

Similar to male except normal sexual dimorphism.

##### Distribution.

Manitoba east to New Brunswick south to Florida west to New Mexico and Utah. Widespread east of the Great Plains (Fig. 23).

##### Biology.

Collected on flowers of Acerspicatum Lam., Alliariapetiolata (M. Beib.) Cavara and Grande, Corema (D. Don) sp., Cornusflorida L., Crataegusmarshallii Eggl., Sassafrasalbidum (Nutt.) Nees, Prunusgracilis Engelm. and A. Gray, Prunusserotina Ehrh., Prunusvirginiana L., Aroniamelanocarpa (Michx.) Elliott, Physocarpusopulifolius (L.) Maxim., Symplocos Jacq. sp., Corus sp., Viburnumcassinoides L., Viburnumlentago L., Viburnumprunifolium L., and Viburnumrafinesquianum Schult. Also collected at Acer L. sap runs. Usually collected in deciduous woods, often near a stream or river, but has also been taken in sphagnum bog. One female has been collected in leaves at base of a hardwood tree.

Known hilltopper. Authors have personally observed specimens flying in a lazy-S-type pattern similar to that of wasps. Recorded flying early March through late July.

##### Remarks.

Desmarest (1848) assigned the name Sphecomyiaboscii to the specimens Latreille used to establish Sphecomyia. The name was forgotten until its rediscovery in Evenhuis and Thompson (1990). We do not assign a neotype as it is uncertain if the series is lost. It is not listed among the MNHN types, nor did the primary author encounter it during a visit to the collection.

COI barcoding recovered two clusters of S.vittata with a maximum barcode divergence barcode of 2.41%. Specimens of both clusters were compared, and no morphological differences were found.

#### Sphecomyia weismani

Taxon classificationAnimaliaDipteraSyrphidae

Moran sp. n.

http://zoobank.org/D935BC53-AAEF-475D-82EB-B4D136D93037

**Figure 3.**
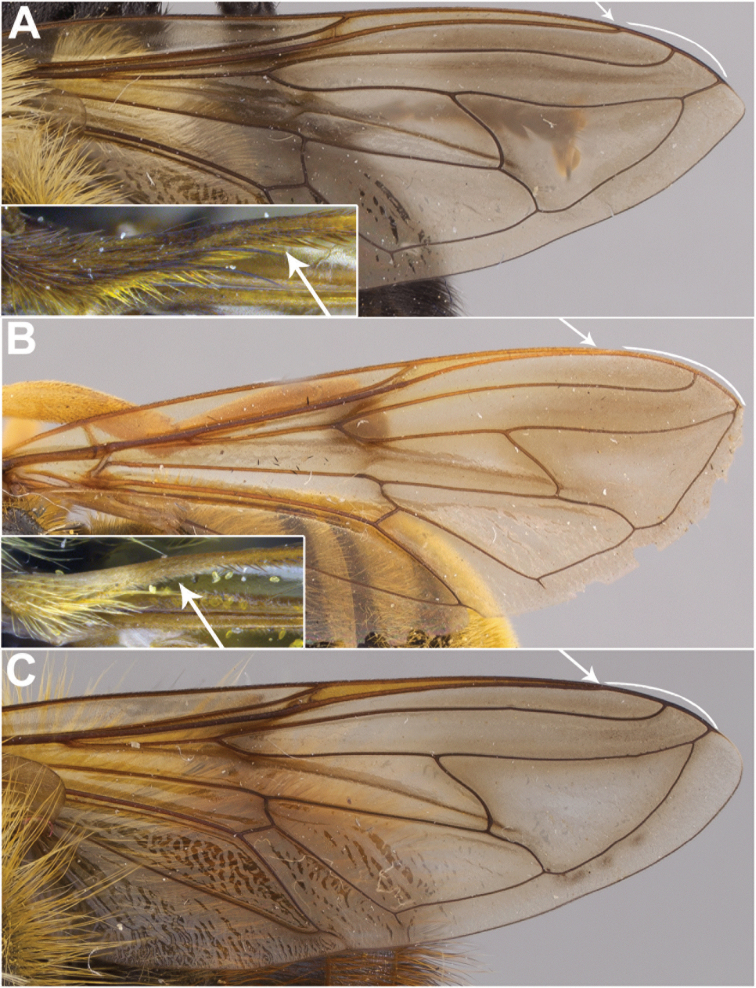
Intersection of vein R_1_ with vein C, distance between apices of veins R_1_ and R_2+3_ and apices of veins R_2+3_ and R_4+5_+M_1_ and setosity of anterior ventral half of vein C before crossvein h. **A***Matsumyia* sp. **B***Sphecomyiaweismani***C***Criorhinabubulcus* (Walker, 1849).

Figs 2O , 3B , 7D , 8D , 9D , 26

##### Type locality.

U.S.A.: Arizona: Greenlee Co., Hannagan Meadows, 33.6392, −109.3263, 2743 m.

##### Types.

*Holotype* male, pinned. Original label: “Hannagan Meadows, 9000’ // Greenlee Co. ARIZ. // I.VII 1966 // R. F. Sternitzky” “CNCDIPTERA 91440”. [1♂, CNC_DIPTERA91440, CNC]

*Paratypes*: U.S.A.: Arizona: Apache Co., Alpine, 33.8481, −109.1431, 2438 m, R.F. Sternitzky, 27.vi.1966, CNC91439 (1♀, CNC); 3.vii.1966, CNC91438 (1♂, CNC); Apache Co., McNary, 34.0719 -109.8550, 2225 m, R.F. Sternitzky, 5.vii.1966, CNC91441 (1♀, CNC); CNC91442 (1♂, USNM); Cochise Co., Parker Canyon, Huachuca Mountains, 31.4278, −110.4519, 1585 m, R.F. Sternitzky, 25.vi.1966, CNC91443 (1♀, CNC).

##### Diagnosis.

It can be confused with *S.columbiana*, *S.cryptica* sp. n., *S.dyari*, *S.hoguei* sp. n., *S.oraria* sp. n., and *S.pattonii* but is easily distinguished by a scutellum with the anterior half pruinose.

##### Description.

**Male.** Body length: 13.6–14.6 mm. Wing length: 9.7–11.0 mm. ***Head.*** Face yellow pruinose with shiny, black, medial vitta extending from oral margin to base of antenna; frons broad, about as long as broad at antenna, two-thirds as broad at vertex as at antenna, bare, with yellow pruinosity along posterior three-fourths; vertex triangular, longer than broad, shiny, with ocellar triangle yellow pilose; postocular border yellow pruinose; postocular and occipital pile yellow; male narrowly dichoptic; antenna black, yellow pilose, length of segments roughly in a 3:3:2 ratio.

***Thorax.*** Sub-shiny black; postpronotum, scutum, scutellum, postalar callus, proepimeron, posterior anepisternum yellow pilose; posterior katepisternum yellow pilose with broadly separated patches; anterior anepimeron yellow pilose; metasternum yellow pilose; postpronotum, anterior half of scutellum, broad posterior margin of anepisternum and dorso-posterior corner of katepisternum yellow pruinose; area between postpronota yellow pruinose, except shiny medially; anepimeron shiny; scutum without pruinose vittae; ventral calypter with long yellow pile.

***Legs.*** Foreleg black, except extreme apex of femur and anterior third of tibia reddish-yellow; fore tarsi slightly broadened; midleg reddish-yellow, except basal four-fifths of femur and last two tarsomeres black; hind leg reddish-yellow, except last two tarsomeres black; legs yellow pilose, except fore tibia, fore tarsi, apex of fore femur black pilose; hind coxa yellow pruinose.

***Wing.*** Hyaline; microtrichia absent from following areas: cell bc; broad basal portion of cell br (before origin of M) and about basal two-fifths of narrower portion of this cell (caudad of spurious vein only); cell bm except apex and narrow anterior and posterior margins of about apical fourth; broad anterior margin of cell cua.

***Abdomen.*** Tergites and sternites shiny to sub-shiny black; with yellow pruinose markings as follows: tergite 1 pruinose along posterior margin; tergite 2 with broad, interrupted, truncate medial band which meets a narrow, uninterrupted posterior band in the posterolateral corners of tergite; tergite 3 with similar medial band, but more narrowly interrupted; pattern on tergite 4 same as tergite 3 except medial band very narrowly or incompletely interrupted; sternite 1 shiny; sternites 2 to 4 almost completely pruinose, with a triangular region of non-pruinosity posteromedially; sternites 6 to 8 pruinose; pile of abdomen and postabdomen yellow.

***Male genitalia.*** Surstylus not elongated, about as long as broad, curving downward ventrally; pile on dorsal and apical fourth of lateral outer surface of surstylus; minute spines on ventral surface, with apical half of lateral inner surface also with spines; basal fourth of the ventral surface of the surstylus produced into a lobe directed anteriorly, with minute pubescence on ventral and lateral inner surface; cerci rounded, with no invagination on posterior border; aedeagus as in Fig. 2O.

##### Female.

Similar to male except normal sexual dimorphism.

##### Distribution.

Arizona (Fig. 26). Known from the Mogollon Rim and Madrean Sky Islands.

##### Biology.

Recorded flying late June through early July.

**Etymology.** The specific epithet honors K. E. Weisman who published a series of four papers on *Sphecomyia* that summarized most of what was previously known about the genus.

## Morphology

**Figure 4.**
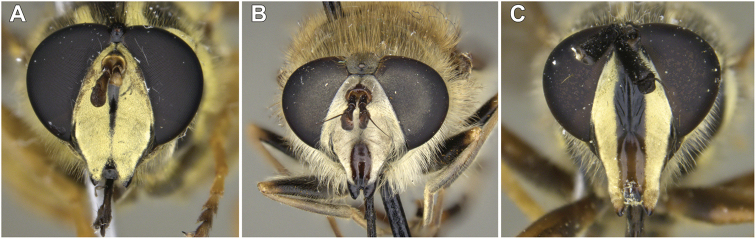
*Sphecomyia* ♂ frontal habitus. **A***Sphecomyiainterrupta***B***Sphecomyiametallica***C***Sphecomyiavespiformis*.

**Figure 5.**
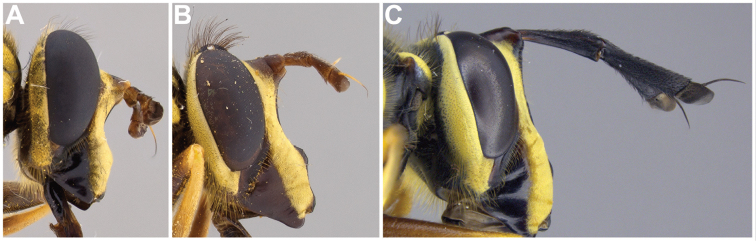
*Sphecomyia* ♂ antenna. **A***Sphecomyiainterrupta***B***Sphecomyiabrevicornis***C***Sphecomyiavittata*

*Sphecomyia* stat. rev. is redefined as the monophyletic unit of species within Criorhinina that possess the following characters: a bare, medial vitta extending ventrally from the oral margin in both sexes (Fig. 4), a bare gena (Fig. 5), a bare katepimeron, a scutellum with at least anterior margin densely pruinose, an anterior ventral half of vein C before crossvein h without setae (Fig. 3B), and a narrow intersection of vein R_1_ with vein C (Fig. 3B). While the combination of characters used to define *Sphecomyia* is unique, the subtribe Criorhinina is rife with homoplasy and the presence of one or more of these character states without all the others should not be taken as an indication a species belongs in *Sphecomyia*.

Like all members of the *Criorhina* group of genera, males of *Sphecomyia* are dichoptic. Holoptic males are only seen in the *Matsumyia* group of genera, although the character is homoplastic within that group. A bare medial vitta extending ventrally from the oral margin is common within the Criorhinina, especially for the female sex. Rare, however, are species in which the character state is present in both the male and female sex. Within the Criorhinina, other than *Sphecomyia*, a bare medial facial vitta is to our knowledge present only in two *Criorhina* species as well as a handful of species which will be placed into the *Matsumyia* group in an upcoming paper. The presence of pile on the gena is homoplastic in all other genera except *Sphecomyia* where it is always absent.

Also completely absent in *Sphecomyia*, a pilose katepimeron is present in almost all true *Criorhina*. However, while uncommon and likely resulting from independent origins, the character state is present in other Criorhinina genera as well. Pruinosity of the scutellum is homoplastic throughout the Criorhinina, with characters states including non-pruinose, weakly pruinose and densely pruinose. However, the character state of an incompletely pruinose scutellum with at least the anterior margin densely pruinose is exclusive to *Sphecomyia*. Only *S.metallica* and *S.interrupta* do not follow this and have a completely pruinose scutellum.

The most reliable character to distinguish between the *Criorhina* group of genera and the *Matsumyia* group of genera is what we define as a narrow intersection of vein R_1_ with vein C as opposed to a broad intersection. In the *Matsumyia* group of genera vein R_1_ is broadly inserted (Fig. 3A), causing the width of the posterior half of cell r_1_ to remain almost unchanged until vein R_1_ abruptly merges with vein C. In the *Criorhina* group of genera, however, vein R_1_ is what we call narrowly inserted (Fig. 3B, C), causing the width of the posterior half of cell r_1_ to rapidly decrease such that vein R_1_ runs alongside vein C as the two veins gradually merge together. A second reliable character aiding in separation of the two groups of genera is the presence or absence of setae on the anterior ventral half of vein C before crossvein h. Members of the *Matsumyia* group of genera are setose on this region (Fig. 3A). Members of the *Criorhina* group of genera are bare (Fig. 3B).

The character state of the distance between apices of veins R_1_ and R_2+3_ longer than distance between apices of veins R_2+3_ and vein R_4+5_+M_1_ (Fig. 3B) is an exclusionary one as the character state in which it is shorter is nearly ubiquitous in the *Matsumyia* group (Fig. 3A) but also found in a subset of species placed in *Criorhina* (Fig. 3C). The character states of erect abdominal pile and appressed abdominal pile are homoplastic to some degree within the Criorhinina. However, erect abdominal pile is the only character state found in *Sphecomyia*.

**Figure 6.**
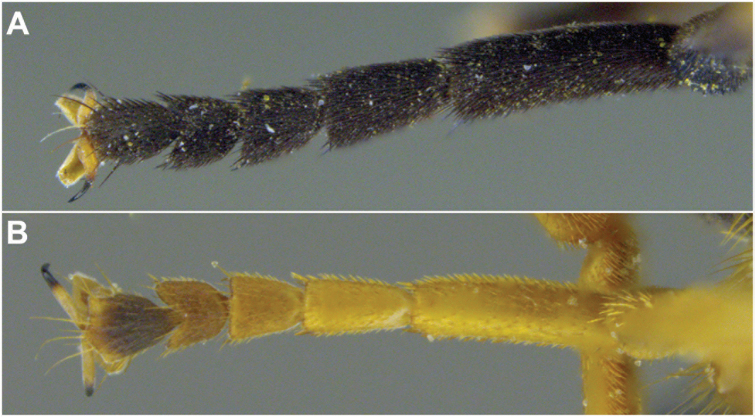
*Sphecomyia* fore tarsi. **A** Slightly Broadened – *Sphecomyiaoraria***B** Not Broadened – *Sphecomyiavittata*.

Morphologically, we recognize three major lineages of *Sphecomyia*. The *vittata* group, composed of the species with pruinose vittae on the scutum, i.e., *S.brevicornis*, *S.interrupta* sp. n., *S.sexfasciata* Moran sp. n., *S.vespiforme*, and *S.vittata*. Secondly, the *pattonii* group comprised of species with broadened fore tarsi (Fig. 6A) and without pruinose vittae on the scutum, i.e., *S.aino* (Stackelberg, 1955), *S.cryptica* Moran sp. n., S.dyari, *S.hoguei* Moran sp. n., *S.oraria* Moran sp. n., *S.pattonii*, *S.pseudosphecomima* Moran sp. n., *S.tsherepanovi* (Violovitsh, 1973), and *S.weismani* Moran sp. n. The third group comprises only one species, *Sphecomyiametallica*, which has a completely pruinose scutum. *S.metallica* shares several characters with the *vittata*group. It has elongated surstyli, with a rounded baso-ventral lobe, reminiscent of the *vittata* group and it lacks the broadened fore tarsi of the *pattonii* group.

A useful character state is pruinosity on sternites 2 to 4 (Fig. 21). Pruinosity patterns on these segments are diagnostic to a species level or almost so. Pattern A (Fig. 21A) is seen in *S.cryptica* and *S.weismani*. Pattern B (Fig. 21B) is seen in *S.dyari* and *S.hoguei*. Pattern C (Fig. 21C) is seen in *S.columbiana*, *S.oraria*, and *S.pattonii*. Pattern D (Fig. 21D) is only in *S.vittata*. Pattern E (Fig. 21E) is only found in *S.vespiformis*. Pattern F (Fig. 21F) is seen in *S.interrupta* and *S.sexfasciata. Sphecomyiabrevicornis* proves to be somewhat of an exception. Northern specimens (i.e. Washington, British Columbia, Idaho, Montana) possess pattern G (Fig. 21G), while Californian specimens possess pattern H (Fig. 21H). In Oregon there is mixture and apparent intermediates of the two states.

Of the two genitalic characters that Weisman (1965) used to distinguish his *vittata* group, i.e. our *Sphecomyia* stat. rev., the first, a banana-shaped phallapodeme (Fig. 2), is, based upon our preliminary investigation, tenatively a synapomorphy shared with *Criorhina* s. str. The second character, a dorsal horn on the basiphallus, is homoplastic throughout Criorhinina.

While Weisman illustrated the aedeagus for each species, our investigation revealed the anterior end of the phallapodeme can vary. Some of this variation is explainable by the length of time genitalia underwent lactic acid clearing as parts of this structure readily lose coloration. Still, there seems to be natural variation in the shape of the phallapodeme such that in the absence of other characters we caution against its use diagnostically or as justification for the erection of new species. The basiphallus and distiphallus, however, do not appear to vary within species.

## COI Gene Tree

DNA barcode data (5' end of the COI) were collected for all 16 morphospecies to test proposed morphological species concepts and to provide a sequence database to assist with identifications of all life stages. Complete barcodes were obtained for all species except *S.cryptica*, *S.oraria*, and *S.pseudosphecomima*. Only fragment C was obtained for *S.cryptica* and *S.pseudosphecomima*, while fragments B and C were obtained for *S.oraria*.

Three major, monophyletic lineages of *Sphecomyia* are resolved in the NJ analysis (Fig. 27) supporting the three morphological groupings. The two species for which only fragment C was obtained, *S.cryptica* and *S.pseudosphecomima*, do not resolve as discrete species in the NJ tree. The two species are lumped with *S.hoguei* and *S.columbiana* respectively. Morphology, however, indicates that this placement is likely an artifact of the short barcode length. *Sphecomyiapseudosphecomima* differs dramatically from *S.columbiana* in that it is silver pruinose on the abdomen and possesses only a single interrupted pruinose band on tergites 2 to 4. *Sphecomyiacolumbiana*, however, is yellow pruinose and possesses two pruinose bands on tergites 2 to 4. For *S.cryptica*, male genitalia as well as pruinosity patterns on sternites 2 to 4 are distinct from *S.hoguei*. The future addition of A and B fragments for these species should enable their clear differentiation through barcodes.

Barcodes revealed that specimens previously identified as *Sphecomyiaaino* resolved into two groupings, one from continental East Palaearctic and a second group from the Japanese and Kuril Islands. Continental *S.aino* are silver-yellow pruinose with entirely, or at least mostly, pale pile on their antennal segments and ocellar triangle. The island-dwelling *S.tsherepanovi* are silver-white pruinose with entirely, or at least mostly, black pile on their antennal segments and ocellar triangle. Additionally, the two populations were found to possess differently shaped dorsal horn on their basiphallus (Fig. 2A L). We argue that these character differences, along with the 3% difference in the DNA barcode between the two taxa, especially considering that the mainland population has little to no variation in COI even across distances greater than 3000 km, are significant enough to warrant separation into two distinct species.

For species for which multiple barcodes were obtained, only one, *S.vittata*, showed high intraspecific variation. Two clusters of *S.vittata* were recovered, resulting in a maximum barcode divergence of 2.41% within the species. Specimens of both clusters were compared, and no morphological differences were found. Two barcodes for *S.brevicornis* were recovered. One from an Alberta specimen and one from a California specimen. The two were 1.3% different, however, neither barcode was complete with the Albertan one missing data at both ends of the sequence and the Californian one missing the middle B fragment. Additional and complete sequences of both the northern and southern morphotypes of *S.brevicornis* are needed to determine whether a gradient exists or whether two discrete clusters are resolved.

Finally, unraveling the relationship of the enigmatic *S.metallica*, the only hairy-bee mimic *Sphecomyia*, with regard to the rest of the genus requires further investigation. The analysis of COI alone placed the species as sister to *Sphecomyia* as a whole. While *S.metallica* shares several characters with the *vittata* group, it is possible these are plesiomorphic and represent shared ancestral traits. Upcoming projects with a multigene phylogeny and target-enrichment data will help with this regard.

## Conclusions

We redefine *Sphecomyia* stat. rev. as the monophyletic unit of Criorhinina containing all species possessing a bare, medial vitta extending ventrally from the oral margin in both sexes, a bare gena, a bare katepimeron, an anterior ventral half of vein C before crossvein h without setae, and a narrow intersection of vein R_1_ with vein C. This redefinition requires the transfer of *Criorhinametallica*, *Criorhinaaino*, and *Criorhinatsherepanovi* to *Sphecomyia*, as they fulfill these requirements, and these new combinations are supported by the COI gene tree. Conversely, removal of three species from *Sphecomyia* and their placement in *Criorhina* is supported by the molecular data and by morphological evidence in their possession of completely pruinose face in the male, a non-pruinose scutellum, appressed pile on the abdomen and a keeled, laterally sclerotized phallapodeme. The species are *Criorhinafusca* (Weisman), comb. n., *C.nasica* (Osburn), comb. n., and *C.occidentalis* (Osburn), comb. n.

**Figure 7.**
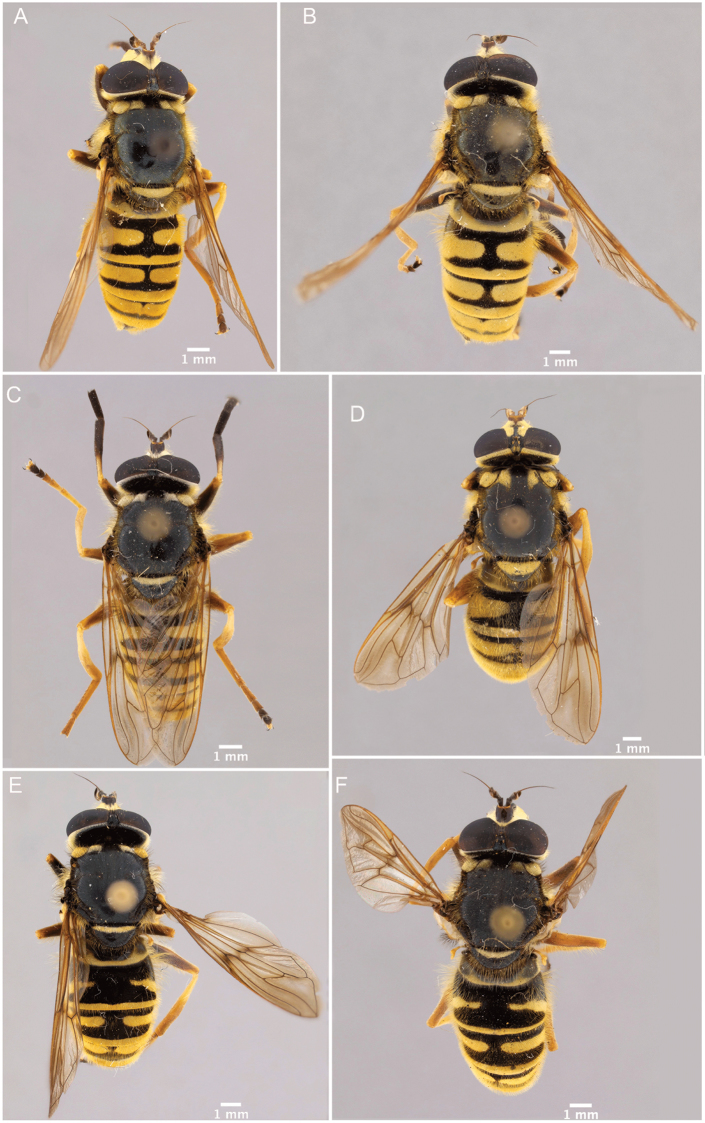
*Sphecomyiapattonii* group dorsal habitus. **A***Sphecomyiadyari***B***Sphecomyiahoguei***C***Sphecomyiacryptica***D***Sphecomyiaweismani***E***Sphecomyiaoraria***F***Sphecomyiapattonii*.

**Figure 8.**
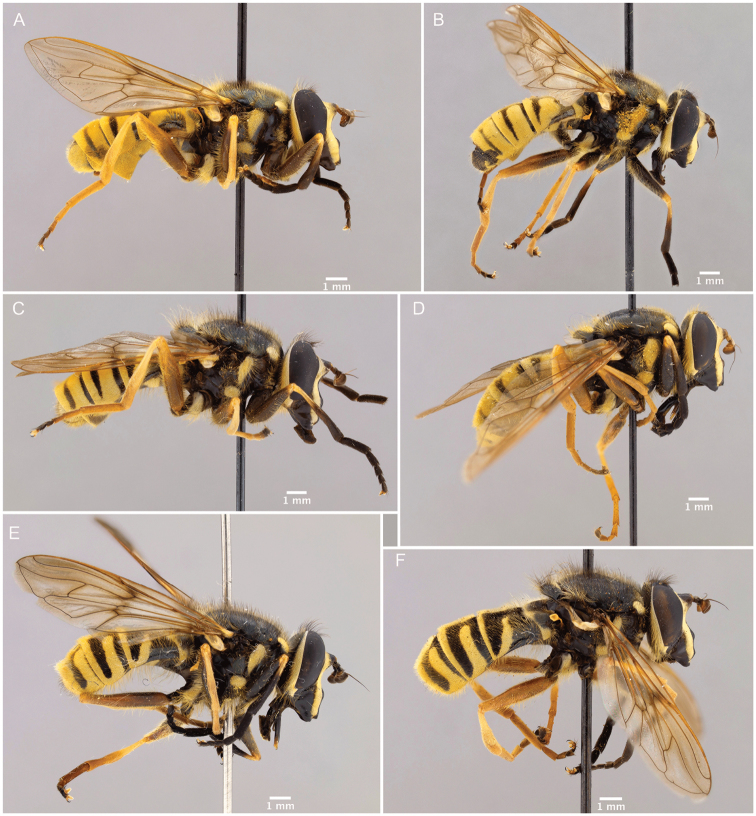
*Sphecomyiapattonii* group lateral habitus. **A***Sphecomyiadyari***B***Sphecomyiahoguei***C***Sphecomyiacryptica***D***Sphecomyiaweismani***E***Sphecomyiaoraria***F***Sphecomyiapattonii*.

**Figure 9.**
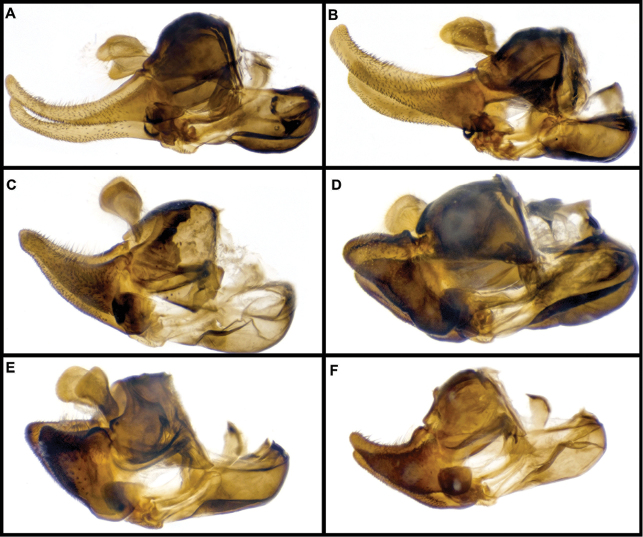
*Sphecomyiapattonii* group male genitalia, lateral view. **A***Sphecomyiadyari***B***Sphecomyiahoguei***C***Sphecomyiacryptica***D***Sphecomyiaweismani***E***Sphecomyiaoraria***F***Sphecomyiapattonii*.

**Figure 10.**
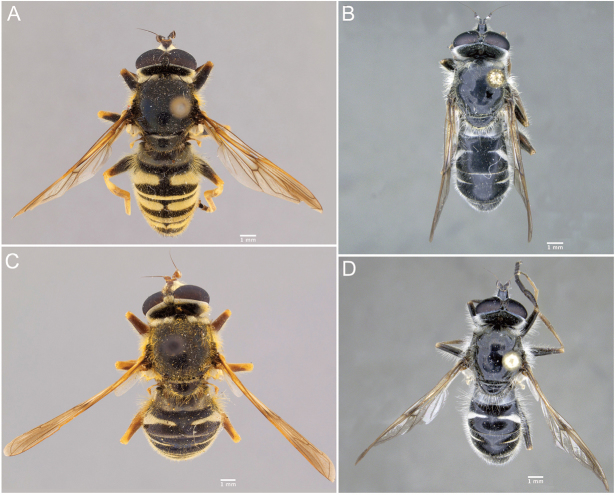
*Sphecomyiapattonii* group (cont.) dorsal habitus. **A***Sphecomyiacolumbiana***B***Sphecomyiaaino***C***Sphecomyiapseudosphecomima***D***Sphecomyiatsherepanovi*

**Figure 11.**
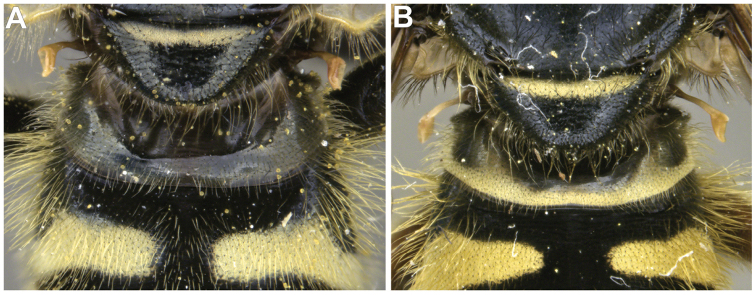
*Sphecomyia* tergite 1. **A***Sphecomyiacolumbiana***B***Sphecomyiapattonii*.

**Figure 12.**
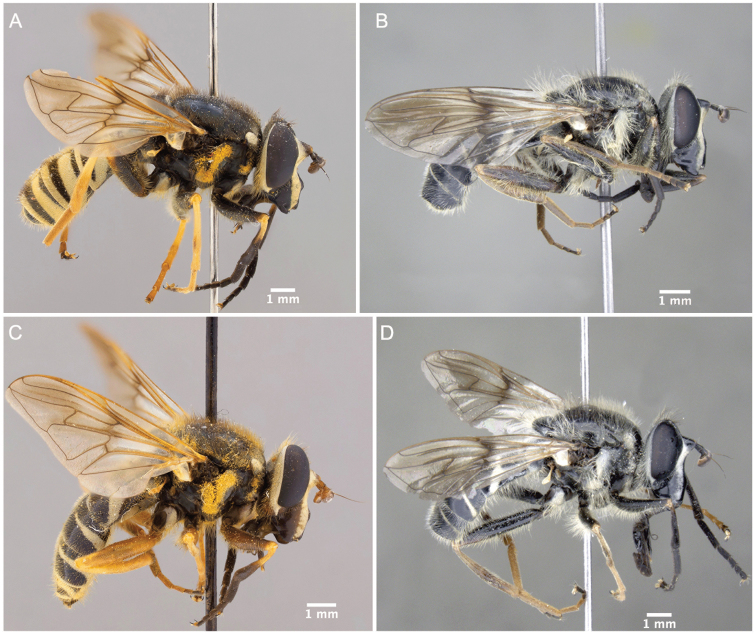
*Sphecomyiapattonii* group (cont.) lateral habitus. **A***Sphecomyiacolumbiana***B***Sphecomyiaaino***C***Sphecomyiapseudosphecomima***D***Sphecomyiatsherepanovi*.

**Figure 13.**
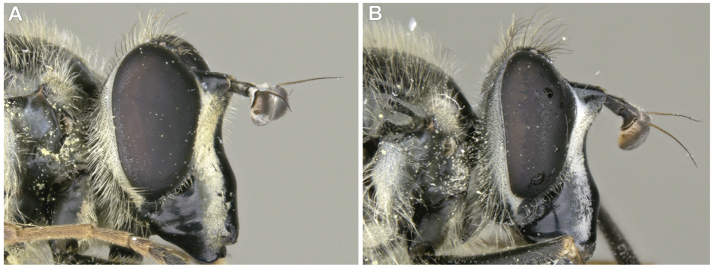
*Sphecomyiapattonii* group (cont.) head in lateral view. **A***Sphecomyiaaino***B***Sphecomyiatsherepanovi*.

**Figure 14.**
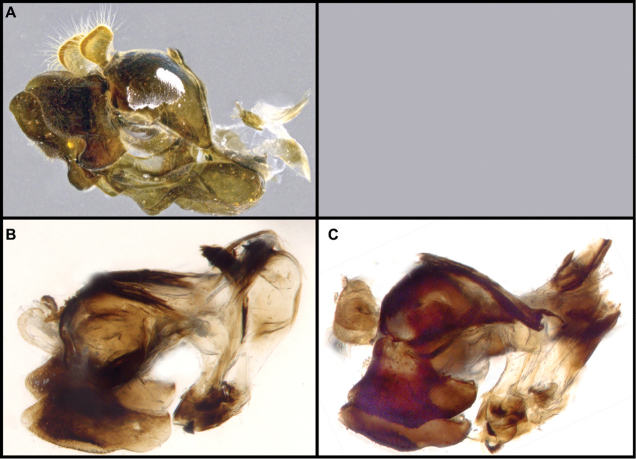
*Sphecomyiapattonii* group (cont.) male genitalia. **A***Sphecomyiacolumbiana***B***Sphecomyiaaino***C***Sphecomyiatsherepanovi*.

**Figure 15.**
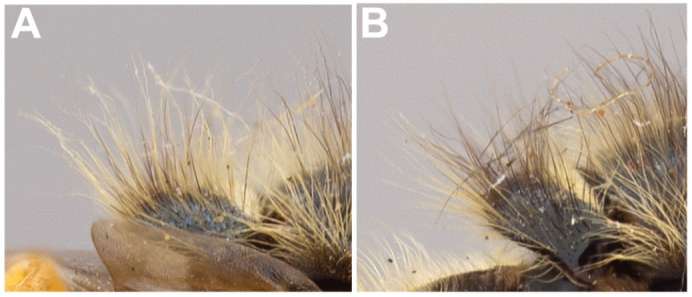
Scutellum pile. **A***Sphecomyiacryptica***B***Sphecomyiaoraria*.

**Figure 16.**
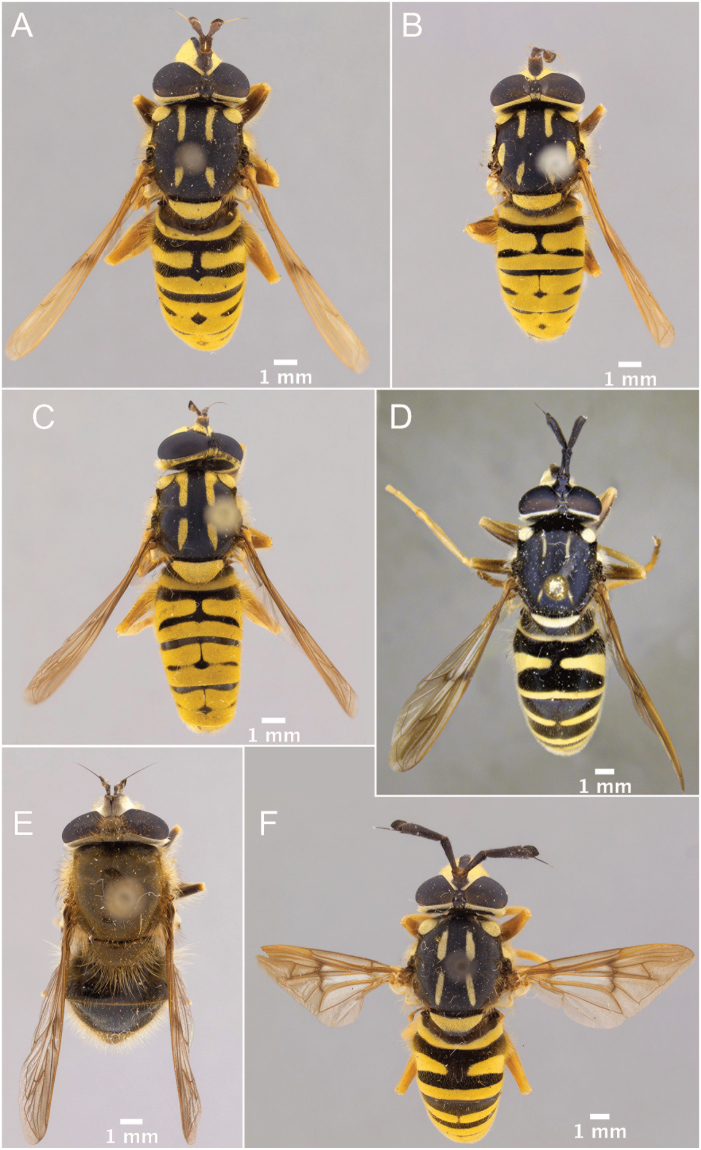
*Sphecomyiavittata* group dorsal habitus. **A***Sphecomyiabrevicornis***B***Sphecomyiasexfasciata***C***Sphecomyiainterrupta***D***Sphecomyiavespiformis***E***Sphecomyiametallica***F***Sphecomyiavittata*,

**Figure 17.**
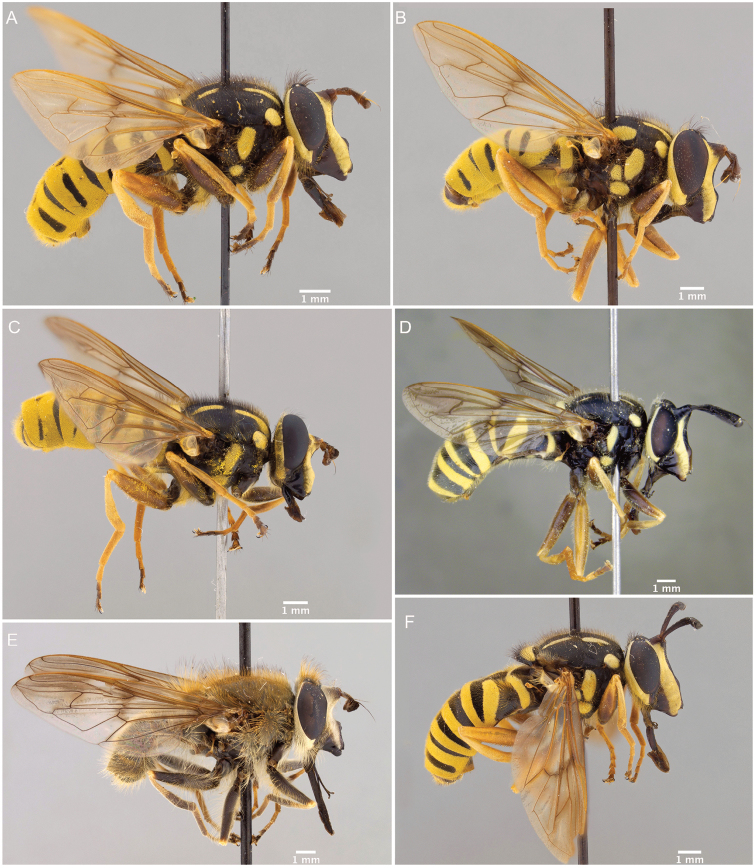
*Sphecomyiavittata* group lateral habitus. **A***Sphecomyiabrevicornis***B***Sphecomyiasexfasciata***C***Sphecomyiainterrupta***D***Sphecomyiavespiformis***E***Sphecomyiametallica***F***Sphecomyiavittata*

**Figure 18.**
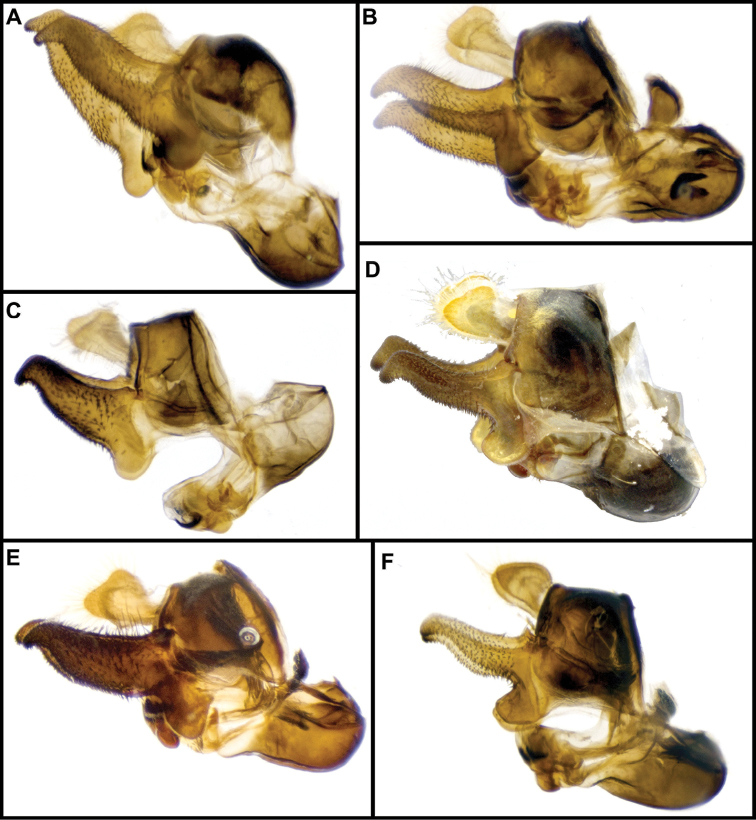
*Sphecomyiavittata* group male genitalia. **A***Sphecomyiabrevicornis***B***Sphecomyiasexfasciata***C***Sphecomyiainterrupta***D***Sphecomyiavespiformis***E***Sphecomyiametallica***F***Sphecomyiavittata*.

**Figure 19.**
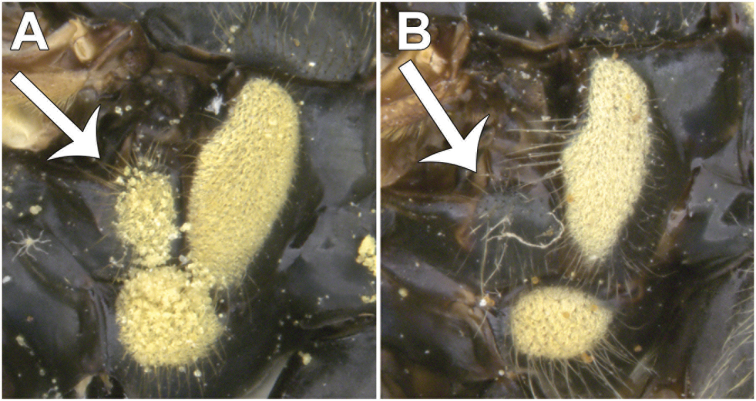
Anepimeron, lateral view. **A***Sphecomyiavittata***B***Sphecomyiavespiformis*.

**Figure 20.**
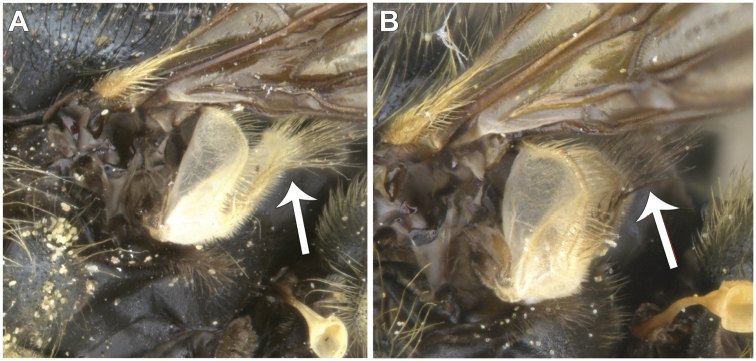
Calypter, lateral view. **A***Sphecomyiaoraria***B***Sphecomyiapattonii*.

**Figure 21.**
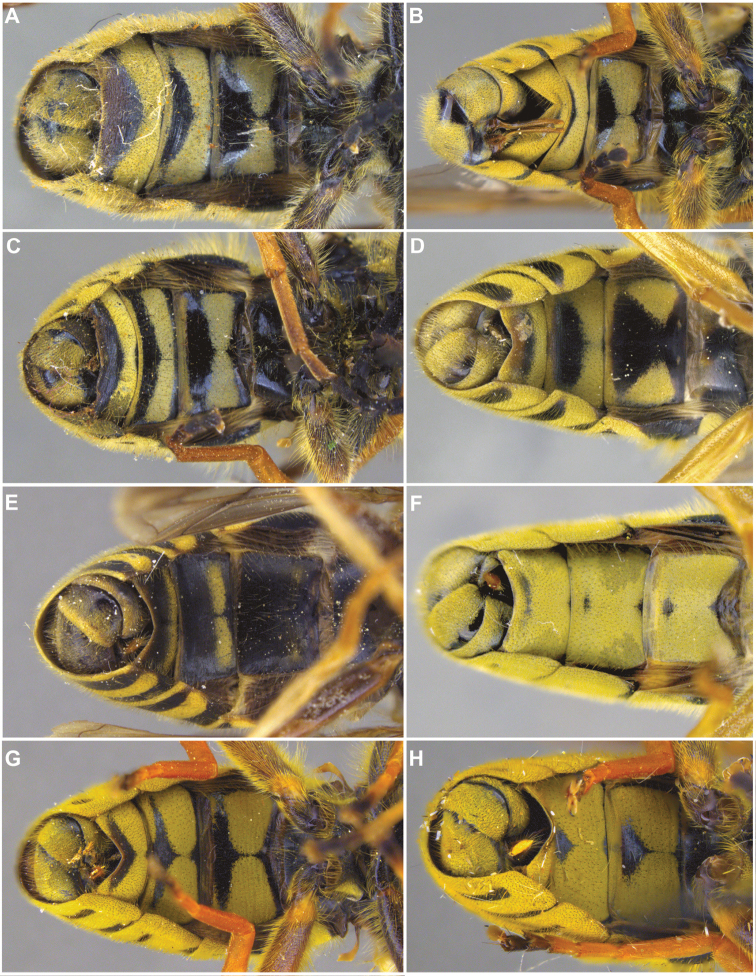
*Sternitepruinosity*. **A***Sphecomyiacryptica***B***Sphecomyiadyari***C***Sphecomyiaoraria***D***Sphecomyiavittata***E***Sphecomyiavespiformis***F***Sphecomyiainterrupta***G***Sphecomyiabrevicornis***H***Sphecomyiabrevicornis*.

**Figure 22.**
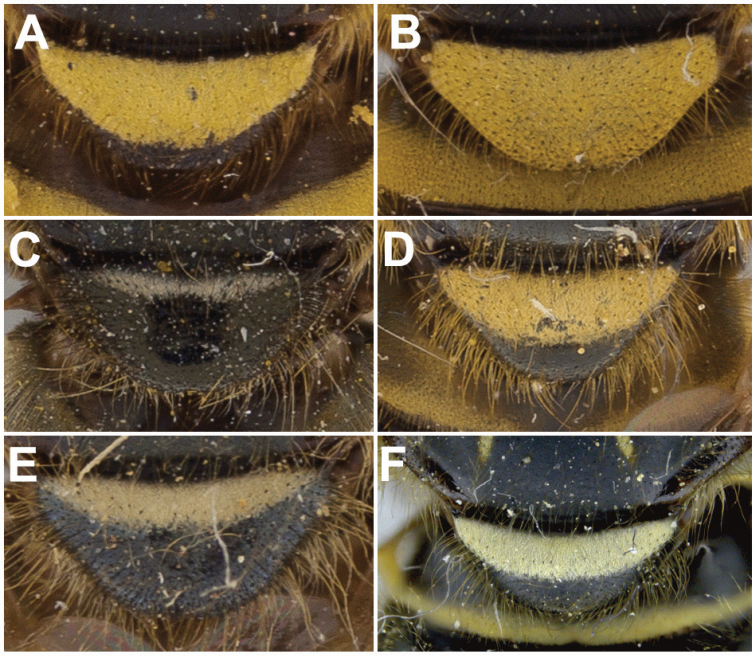
Scutellum pruinosity. **A***Sphecomyiabrevicornis***B***Sphecomyiainterrupta***C***Sphecomyiacolumbiana***D***Sphecomyiaweismani***E***Sphecomyiacryptica***F***Sphecomyiavespiformis*

**Figure 23.**
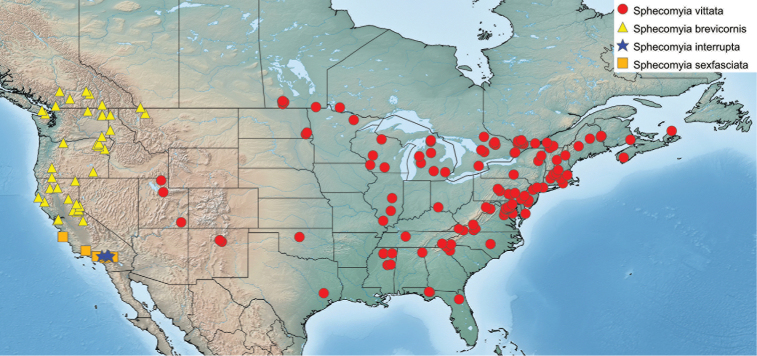
*Sphecomyiavittata* group distribution.

**Figure 24.**
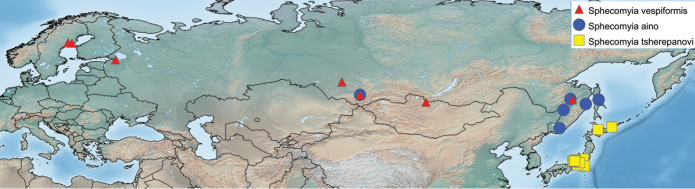
Old World *Sphecomyia* distribution.

**Figure 25.**
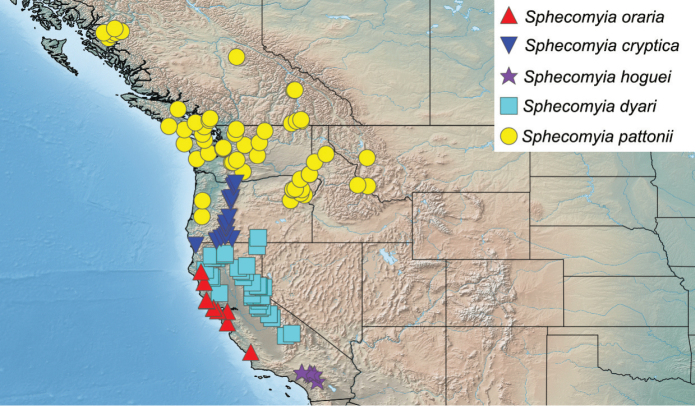
*Sphecomyiapattonii* group distribution.

**Figure 26.**
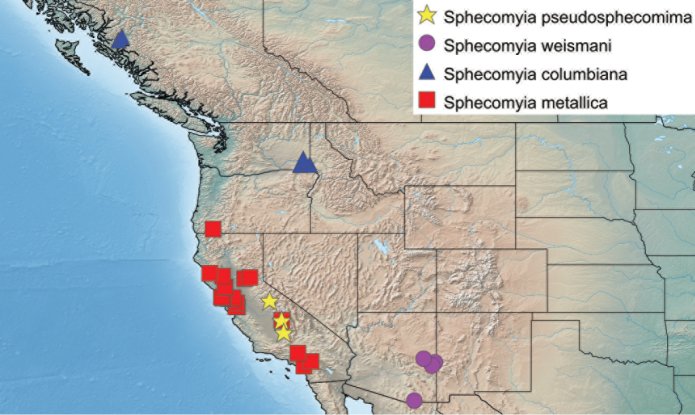
*Sphecomyiapattonii* group (cont.) and *Sphecomyiametallica* distribution.

**Figure 27.**
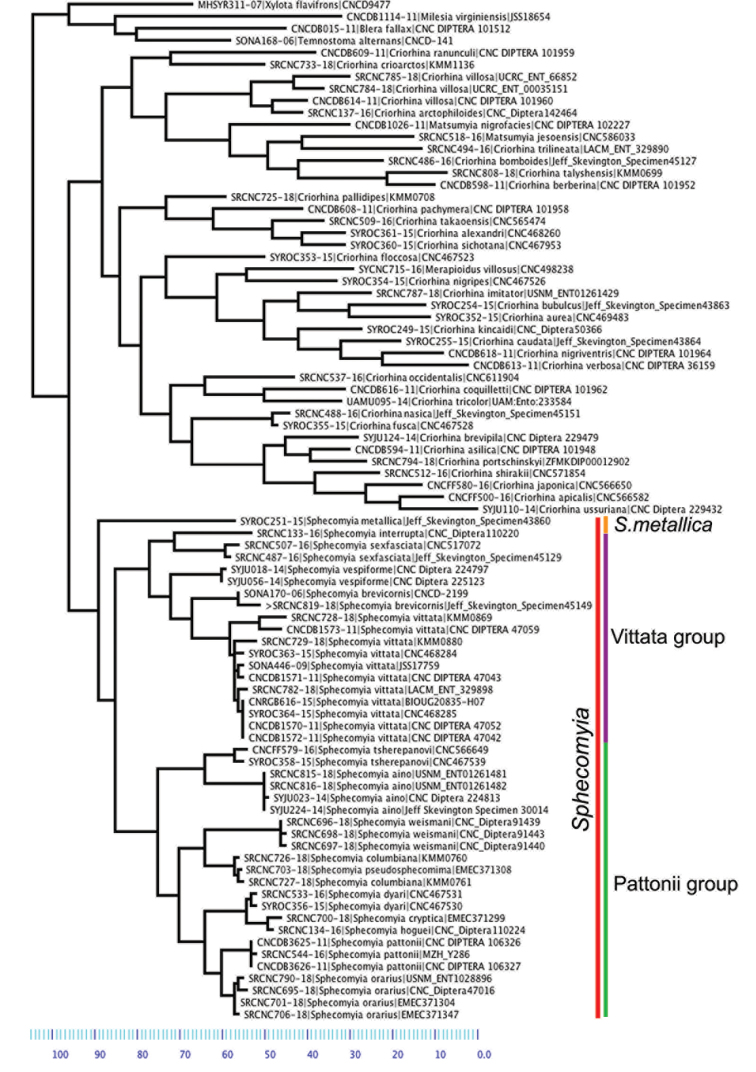
Neighbor Joining Tree.

## Supplementary Material

XML Treatment for Sphecomyia

XML Treatment for Sphecomyia aino

XML Treatment for Sphecomyia brevicornis

XML Treatment for Sphecomyia columbiana

XML Treatment for Sphecomyia cryptica

XML Treatment for Sphecomyia dyari

XML Treatment for Sphecomyia hoguei

XML Treatment for Sphecomyia interrupta

XML Treatment for Sphecomyia metallica

XML Treatment for Sphecomyia oraria

XML Treatment for Sphecomyia pattonii

XML Treatment for Sphecomyia pseudosphecomima

XML Treatment for Sphecomyia sexfasciata

XML Treatment for Sphecomyia tsherepanovi

XML Treatment for Sphecomyia vespiformis

XML Treatment for Sphecomyia vittata

XML Treatment for Sphecomyia weismani

## Supplementary materials

Supplementary material 1 *Sphecomyia* specimen data Data type: specimen data This dataset is made available under the Open Database License (http://opendatacommons.org/licenses/odbl/1.0/). The Open Database License (ODbL) is a license agreement intended to allow users to freely share, modify, and use this Dataset while maintaining this same freedom for others, provided that the original source and author(s) are credited.Kevin M. Moran, Jeffrey H. Skevington
